# Organic phosphorescent scintillation from copolymers by X-ray irradiation

**DOI:** 10.1038/s41467-022-31554-3

**Published:** 2022-07-09

**Authors:** Nan Gan, Xin Zou, Mengyang Dong, Yanze Wang, Xiao Wang, Anqi Lv, Zhicheng Song, Yuanyuan Zhang, Wenqi Gong, Zhu Zhao, Ziyang Wang, Zixing Zhou, Huili Ma, Xiaowang Liu, Qiushui Chen, Huifang Shi, Huanghao Yang, Long Gu, Zhongfu An, Wei Huang

**Affiliations:** 1 Frontiers Science Center for Flexible Electronics (FSCFE), MIIT Key Laboratory of Flexible Electronics (KLoFE), Northwestern Polytechnical University, Xi’an, 710072 P.R. China; 2 Key Laboratory of Flexible Electronics (KLoFE) & Institute of Advanced Materials (IAM), Nanjing Tech University (NanjingTech), 30 South Puzhu Road, Nanjing, 211816 P.R. China; 3 MOE Key Laboratory for Analytical Science of Food Safety and Biology, State Key Laboratory of Photocatalysis on Energy and Environment, College of Chemistry, Fuzhou University, Fuzhou, 350108 P.R. China

**Keywords:** Optical materials and structures, Materials for optics

## Abstract

Scintillators that exhibit X-ray-excited luminescence have great potential in radiation detection, X-ray imaging, radiotherapy, and non-destructive testing. However, most reported scintillators are limited to inorganic or organic crystal materials, which have some obstacles in repeatability and processability. Here we present a facile strategy to achieve the X-ray-excited organic phosphorescent scintillation from amorphous copolymers through the copolymerization of the bromine-substituted chromophores and acrylic acid. These polymeric scintillators exhibit efficient X-ray responsibility and decent phosphorescent quantum yield up to 51.4% under ambient conditions. The universality of the design principle was further confirmed by a series of copolymers with multi-color radioluminescence ranging from green to orange-red. Moreover, we demonstrated their potential application in X-ray radiography. This finding not only outlines a feasible principle to develop X-ray responsive phosphorescent polymers, but also expands the potential applications of polymer materials with phosphorescence features.

## Abstract (web summary)

The authors achieved the X-ray-excited organic phosphorescent scintillation from copolymers through copolymerization of bromine-substituted chromophores and acrylic acid and demonstrated their potential application in X-ray radiography.

## Introduction

Scintillators, as a category of X-ray responsive luminescent materials, have recently attracted growing attention due to their exceptional ability that converts high-energy X-rays into low-energy ultraviolet or visible photons^1^, which show great potential for various practical applications, such as radiation detection, X-ray imaging, radiotherapy, and non-destructive testing^2–6^. Conventional scintillators could be roughly grouped into inorganic and organic scintillating materials. Thereinto, inorganic scintillators, mainly available as bulk crystals grown at high temperatures are based on high-atomic-number (*Z*) metal elements to enhance the interaction probability with ionizing radiation for strong X-ray absorption^7–10^. Although these inorganic scintillators exhibit excellent properties like strong stopping power, high light output, and low limit of detection, there remain great challenges in processability, modifiability, and production cost^11,12^. In contrast with inorganic scintillators, organic counterparts including organic crystals, organic liquids, and plastic scintillators have emerged as promising scintillating materials owing to their inherent merits in terms of affordable raw materials, easy modification and processing, and large-area fabrication^13,14^. However, organic dyes with limited effective atomic number result in weak X-ray absorption^15–17^. Besides, most of the reported organic scintillators are fluorescent materials. In this situation, due to spin statistics, only a small portion of singlet excitons could be utilized for radioluminescence. While most triplet excitons are dissipated via non-radiative decay owing to their dark state characteristic in purely organic chromophores.

In contrast to fluorophores, organic phosphors with phosphorescence emission can make the most of the excitons (~100%) for efficient luminescence owing to their outstanding ability to harvest both singlet and triplet excitons^18–24^. To achieve efficient phosphorescence from purely organic materials, recently, great efforts have been devoted to suppressing the non-radiative decay of triplet excitons via constructing a rigid microenvironment and improving the intersystem crossing (ISC) between the singlet and triplet excited states by incorporating heavy halogen atoms or aromatic carbonyl groups into organic chromophores, respectively^25–30^. Among these universal design strategies, such as host-guest doping^31–33^, polymerization^34–36^, supramolecular assembling^24,37,38^, and so forth^39^, crystal engineering has played an essential role in achieving organic phosphorescence with high efficiency at room temperature^40^. Based on this, our group recently realized highly efficient X-ray-excited luminescence in a series of organic crystalline materials with bright triplet excitons^41^. Despite the superior X-ray scintillation performance of the organic phosphorescent materials, the intrinsic obstacles of the crystals in integration and processability for device applications would greatly hinder the development of organic X-ray scintillating materials. Compared with crystal scintillators, polymers may be one type of the promising candidates since their intriguing advantages, such as reproducibility, processability, film-forming ability, flexibility, and so on^42^. However, to the best of our knowledge, there is no report on purely organic phosphorescent polymers for radioluminescence under X-ray irradiation.

**Fig. 1:**
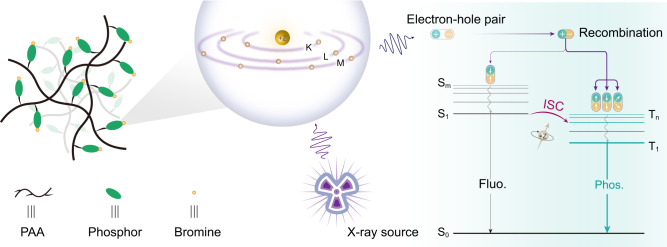
Rational design of amorphous copolymers for organic phosphorescent scintillation and the related emissive processes under X-ray irradiation. After X-ray irradiates the polymers and ejects the inner electrons of the halogen atoms, plenty of electrons and holes are produced. Then the recombination of holes and electrons produces singlet and triplet excitons. Among them, radiative transitions of the excitons generate radioluminescence under ambient conditions. Fluo. and Phos. refer to fluorescence and phosphorescence, respectively.

In this work, we present a facile strategy to achieve the X-ray excited phosphorescent radioluminescence from amorphous polymers through the simple radical copolymerization of the heavy halogen atoms-substituted chromophores and acrylic acids. Generally, the absorption of X-ray and the decay of excited states of materials play a crucial role in obtaining the phosphorescent radioluminescence under X-ray irradiation (Fig. 1). The acrylic acid was chosen because the numerous carboxyl groups in polyacrylic acid (PAA) could restrict the molecular motions of chromophores with multiple inter/intra-molecular hydrogen bonds, thus reducing non-radiative transition for favoring phosphorescence generation. Meanwhile, the selection of bromine (Br) atoms could enhance the spin-orbit coupling (SOC) for efficiently populating triplet excitons owing to the heavy atom effect. More significantly, the high *Z* of the bromine atom is beneficial for X-ray absorption and transition during X-ray irradiation (Supplementary Fig. 1). Besides, luminescence efficiency is one of the important factors for indirect X-ray detection. Given this, organic dyes with high photoluminescence quantum yields are welcome.

## Results

### Synthesis and photophysical properties of the copolymer PBBr

As a proof-of-concept experiment, we first synthesized a copolymer of PBBr-50 by free-radical copolymerization of acrylic acid (AA) and monomer 4-(allyloxy)−4′-bromo-1,1′-biphenyl (BBr) with 2-azoisobutyronitrile (AIBN) as an initiator, wherein the BBr has been frequently used as a high luminescence efficiency phosphorescent chromophore^43–46^ (Supplementary Methods, Supplementary Fig. 2). The molar feed ratio of BBr/AA was 1/50 for PBBr-50. The chemical structures of the target monomer and PBBr copolymer were fully confirmed by nuclear magnetic resonance (^1^H and ^13^C NMR) spectroscopy. And the polymer molecular weight and polydispersity (PDI) were characterized by aqueous gel permeation chromatography (GPC) (Supplementary Figs. 3–18 and Supplementary Table 1). Their morphologies were also characterized by transmission electron microscopes (TEM, Supplementary Figs. 19–28 and Supplementary Tables 2–8), showing the uniform film structure features. As predicted, the prepared polymer material of PBBr-50 displayed visualized highly efficient phosphorescence under ambient conditions (Supplementary Movie 1).

**Fig. 2:**
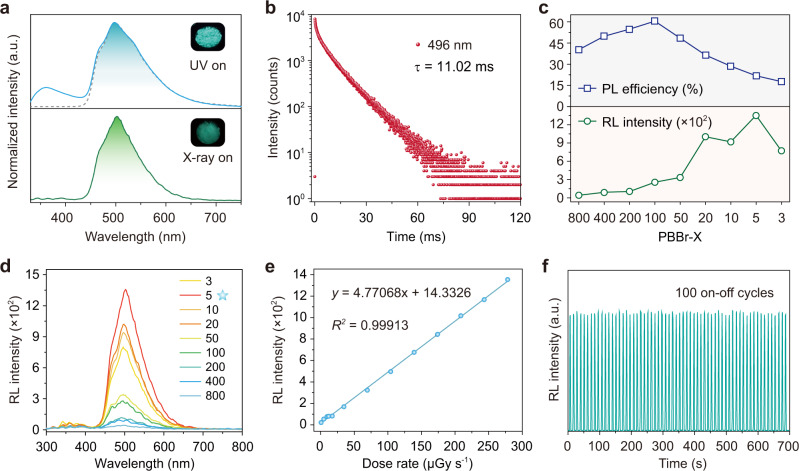
Photophysical properties of the PBBr copolymers by X-ray irradiation under ambient conditions. **a** Normalized steady-state photoluminescence (PL, blue line) and phosphorescence (gray line) spectra of the PBBr-50 film under UV light excitation, as well as radioluminescence (RL) spectrum (green line) at a dose rate of 278 μGy s^−1^. Insets are the corresponding ultraviolet (UV) light- and X-ray-excited photographs. **b** A lifetime decay curve of emission band at 496 nm for PBBr-50 film. **c** PL efficiency and RL intensity variation of copolymer films from PBBr-3 to PBBr-800. **d** RL spectra of different PBBrs at a dose rate of 278 μGy  s^−1^. Note: the examination was quantitative for each group. **e** RL measurements of the PBBr-5 film as a function of dose rate in the range of 0.688 to 278 μGy s^−1^. **f** Emission photostability of the PBBr-5 film at 500 nm under repeated on-off cycles of X-ray at a dose rate of 278 μGy s^−1^.

We then systematically investigated the photophysical properties of the PBBr-50 polymer film by steady-state photoluminescence (PL) and phosphorescence spectra under ambient conditions (Fig. 2a). Because of the introduction of the bromine atoms, the copolymer PBBr-50 exhibited distinct dual-emission with a weak emission band at around 360 nm and an intense emission band at around 496 nm upon excitation by 310 nm ultraviolet (UV) light. After a delay time of 8 ms, there is only one phosphorescent emission band with a maximum peak at 496 nm, of which the profiles remained constant as excitation wavelengths change from 220 to 340 nm (Supplementary Fig. 29). Impressively, under X-ray irradiation, a bright green radioluminescence with a prominent band around 500 nm was observed from the PBBr-50 film, which was consistent with the corresponding phosphorescent spectrum.

To further confirm the feature of the emission bands, we performed the lifetime decay profiles of copolymer PBBr-50 at room temperature. As shown in Fig. 2b and Supplementary Fig. 30, we found that the weak emission band at about 360 nm showed a short lifetime of 4.45 ns, suggesting fluorescence behavior. Nevertheless, the green emission peak of 496 nm exhibited a long lifetime up to 11.02 ms under ambient conditions, demonstrating a phosphorescence nature. Notably, compared with the photoluminescent behaviors of the copolymer excited by UV light, we found that the phosphorescence proportion of the copolymer PBBr-50 was enhanced dramatically in the radioluminescence (RL) spectrum under X-ray irradiation. Meanwhile, the integral area ratio of phosphorescence-to-fluorescence displayed an increase of 8.6 times from 5.63 to 48.89 (Fig. 2a and Supplementary Fig. 31a). These results indicate that the excitons were efficiently harvested for the phosphorescence during radioluminescence processes.

### Effect of variation in molar feed ratio on RL and PL properties of the copolymers

Notably, the introduction of the bromine atoms on organic chromophores plays a crucial role in manipulating photoluminescence and radioluminescence due to its unique heavy atom effect. Therefore, we further prepared a series of PBBr copolymers with various molar feed ratios of monomer BBr/AA ranging from 1/3 to 1/800 to systematically explore the relationship between the content of the bromine atoms and X-ray responsive luminescent properties. As increasing the molar feed ratios from 1/3 to 1/800, these copolymers exhibited a similar photoluminescence behavior under UV light excitation, along with a slight increase in phosphorescent lifetimes from 9.22 to 11.76 ms (Supplementary Fig. 30 and Supplementary Table 9). However, the PL quantum yields and radio-luminescent intensity of the copolymers showed a significant difference (Fig. 2c, d). With the increase of relative concentrations of monomer BBr from PBBr-800 to PBBr-3, the PL quantum yields first exhibited a growing trend, with the highest PL efficiency reaching up to 60.5% (*Φ*_Phos_. = 51.4%) from PBBr-100. And then, the data decreased dramatically from 60.5% to 17.7%, which might be attributed to the quenching of triplet excitons resulting from the dense distribution of the aromatic chromophores in the polymer film (Supplementary Table 10)^47–49^. In contrast, with the concentrations of BBr increasing from 1/800 to 1/3, RL intensities of these copolymers at 500 nm demonstrated an increasing trend overall, and up to the maximum value when the molar feed ratio of BBr/AA is 1/5. These findings indicated the appropriate content of bromine atom-containing monomers is a key factor for achieving efficient phosphorescence radioluminescence in copolymers. This speculation was further confirmed by elementary mapping and energy-dispersive spectroscopy (EDS), suggesting that the Br atoms were uniformly distributed throughout the polymeric films, and the related contents of the Br atoms increased gradually with the molar feed ratios of BBr/AA ranging from 1/800 to 1/3 (Supplementary Figs. 19–27 and Supplementary Tables 3–8). Considering the decent radioluminescence performance of the PBBr-5 film compared with other PBBrs copolymers, we then took this sample as a model to investigate the responsivity and photostability as a function of the X-ray dosage. As shown in Fig. 2e and Supplementary Fig. 31b, the RL intensities were a linear response to the X-ray dose rate ranging from 0.688 to 278 μGy s^−1^, displaying a good responsivity. Meanwhile, it was found that the emission intensity of the copolymer at 500 nm was very stable even under a high dose rate of repeated X-ray (278 μGy s^−1^) excitation for 100 on-off circles (Fig. 2f). Besides, we also studied the radio-stability of PBBr-5 film. After exposure to continuous radiation of X-ray for 30 min, the radioluminescence intensity could remain basically stable (Supplementary Fig. 32). These decent radioluminescence behaviors and good processability of the PBBr-5 copolymer make it possible to be applied for X-ray detection.

### Proposed mechanism for radioluminescence in amorphous copolymers

**Fig. 3:**
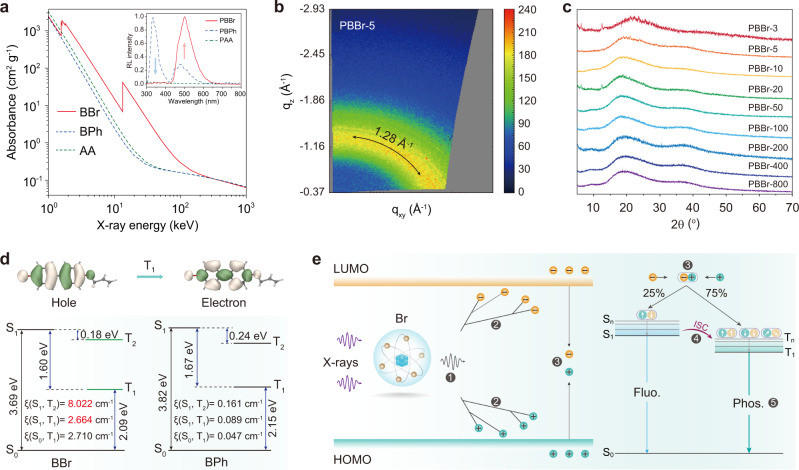
Mechanism of phosphorescent scintillation from organic copolymers under X-ray irradiation. **a** X-ray absorption spectra of the BBr, BPh, and AA monomers. The insert shows the normalized radioluminescence (RL) intensity of the PBBr, PBBh, and PAA polymers at a dose rate of 278 μGy s^−1^. **b** WAXS pattern of PBBr-5 polymer film. **c** PXRD patterns of the copolymers from PBBr-3 to PBBr-800. **d** Natural transition orbitals (NTOs) for the lowest triplet state of BBr, and calculated excitation energies and spin-orbit coupling (SOC) constants (*ξ*) of the BBr and BPh monomers. **e** Proposed mechanism of radioluminescence for amorphous copolymers. After X-ray irradiation, the electron in the inner shells is excited by high-energy X-ray photons and ejected out of the Br atom. Then, the high-energy electrons generate lots of secondary electrons by interacting with other atoms in the polymer. The generated electrons and holes are rapidly thermally dissipated and gradually accumulate at the lowest unoccupied molecular orbital (LUMO) and highest occupied molecular orbital (HOMO) of the organic phosphors, respectively. Eventually, the electrons and holes recombine to form excited states, generating singlet and triplet excitons in a ratio of 1:3, which produces fluorescence (Fluo.) and phosphorescence (Phos.) via the radiative decay processes, respectively.

To probe the underlying mechanism of phosphorescent scintillation by X-ray irradiation in organic copolymers, we designed and synthesized other two control polymers PAA and PBPh. In monomer BPh, the bromine atom was substituted by a hydrogen atom (Supplementary Figs. 6–8, 18–19 and Supplementary Table 2). As shown in Fig. 3a, we first made a comparison on the X-ray absorption coefficients of monomers BBr (*Z*_max_ = 35, *K*_α_ = 13.5 keV), BPh (*Z*_max_ = 8, *K*_α_ = 0.525 keV), and acrylic acid (*Z*_max_ = 8, *K*_α_ = 0.525 keV), respectively^50^. It was found that bromine atom-containing monomer BBr displayed a larger X-ray absorption coefficient than the other two monomers within the energy region from 1 to 1,000 keV, confirming the effect of bromine atoms in effective absorbing X-ray photons. This trend matches well with their corresponding radioluminescence experiments under the irradiation of a dose rate of 278 μGy s^−1^ (insert in Fig. 3a). Compared with the control polymers PAA and PBPh, the phosphorescent proportion of copolymer PBBr exhibited a remarkable enhancement in the X-ray-excited luminescence spectrum. Meanwhile, the PAA film was hardly observed any obvious optical signals from the RL spectrum due to the absence of X-ray responsive chromophores. These results suggest that the bromine atoms play a vital role in the process of X-ray absorption to populate triplet excitons for generating phosphorescent radioluminescence.

To further reveal the origination of photoluminescence and radioluminescence of the copolymers, we first performed Grazing-incidence wide-angle X-ray scattering (GI-WAXS) experiments of various PBBr copolymers. Obviously, only two broad scattering bands at around 1.28 Å (18.21°) and 2.47 Å (35.80°) attributed to the characteristic scattering bands of PAA can be observed^34^. There were no other π–π interactions from the aggregates detected (Fig. 3b and Supplementary Fig. 33). This indicated that the PL and RL feature derived from isolated monomer BBr in PAA film. Notably, we found that the photophysical properties of the monomer BBr in dilute dichloromethane (DCM) solution (2 × 10^−5^ M) at 77 K as well as doped in polymethyl methacrylate (PMMA, 1 wt%) under ambient conditions are similar to those of copolymer PBBr at room temperature (Supplementary Fig. 34). Therefore, we concluded that the green emission of the PBBr copolymers under X-ray irradiation originated from the phosphorescent emission of the isolated BBr chromophores. Meanwhile, the powder X-ray diffraction (PXRD) patterns further demonstrated the amorphous nature of PBBrs (Fig. 3c). To gain a deep insight into the luminescence mechanism, the time-dependent density functional theory (TDDFT) calculations based on monomers BBr and BPh were carried out (Fig. 3d and Supplementary Fig. 35). It is worth noting that the spin-orbit coupling constants (*ξ*) of S_1_ to T_n_ for monomer BBr (S_1_-T_2_, 8.022 cm^−1^; S_1_-T_1_, 2.664 cm^−1^) were larger than those of the BPh (S_1_-T_2_, 0.161 cm^−1^; S_1_-T_1_, 0.089 cm^−1^), indicating a highly efficient heavy-atom-mediated ISC process for phosphorescence generation. Furthermore, it was found that the calculated phosphorescent radiative decay rate constant *k*_p_ of PBBr-5 (17.8 s^−1^) was indeed larger than that of the PBPh-5 (0.035 s^−1^) with over 500 times (Supplementary Fig. 36 and Supplementary Tables 11–12). These results elucidate the incorporation of heavy atoms on organic chromophore not only improves the ability of X-ray absorption of materials, but also promotes ISC to populate the triplet excitons for effective radioluminescence. In addition, we also investigated the effect of iodide or more bromide substitution on the scintillation performance of the resulting copolymers (Supplementary Figs. 37–42 and 45–47 and Supplementary Tables 13–15). From the results, we concluded that the selection of the chromophores with high Z elements, efficient SOC, and high luminescence efficiency, as well as the construction of a rigid polymer matrix are favorable to construct efficient polymeric phosphorescence radioluminescence materials.

Taken together, we proposed a plausible mechanism for phosphorescent radioluminescence of the amorphous copolymers. As shown in Fig. 3e, owing to the presence of heavy atoms, the PBBr copolymers could efficiently absorb the high-energy X-ray, resulting in numerous electrons ejected from the inner shell of atoms via the photoelectric effect. Subsequently, after electron interaction processes, the electrons and holes recombine to populate the singlet and triplet excitons in a ratio of 1:3, according to the spin statistics. Then the fluorescence and phosphorescence of scintillating material were generated through the radiative transition of singlet and triplet excitons, respectively. Notably, owing to the synergy of the efficient X-rays absorptivity and the strong SOC of the BBr monomers with the construction of a rigid microenvironment by PAA polymer segments, the PBBr copolymers displayed more efficient phosphorescence under the X-ray irradiation than that excited by UV light under ambient conditions (Supplementary Fig. 1).

### The general strategy for synthesizing the copolymers with colorful phosphorescent radioluminescence

**Fig. 4:**
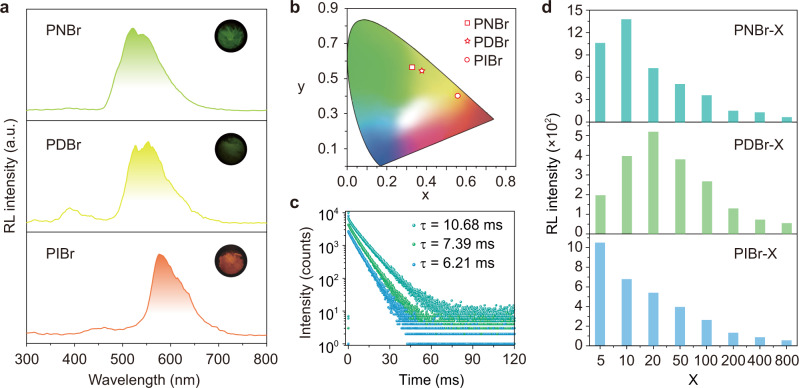
Colorful phosphorescent radioluminescence in organic copolymers under X-ray irradiation. **a** Normalized radioluminescence spectra and the related photographs of PNBr, PDBr, and PIBr copolymer films under the irradiation of a dose rate of 278 μGy s^−1^, respectively. **b** CIE chromaticity coordinate diagram of the radioluminescence color of the copolymers. **c** Lifetime decay curves of emission bands at 546, 555, and 575 nm for PNBr, PDBr, and PIBr copolymer films, respectively, under UV light excitation. **d** Radioluminescence (RL) intensity variation of PNBr, PDBr, and PIBr copolymer films with different molar feed ratios of two monomers by X-ray irradiation at a dose rate of 278 μGy s^−1^. Note that the examination was quantitative for each group.

To validate the generality of our strategy, we designed and synthesized other three copolymers containing different Br-substituted monomers^51^, namely PNBr, PDBr, and PIBr, respectively (Supplementary Figs. 2 and 9–17). Impressively, all the copolymer films displayed obvious X-ray-excited luminescence with maximum emission peaks at 522 nm for PNBr film, 555 nm for PDBr film, and 580 nm for PIBr film, respectively, demonstrating the multiple emission colors spanning from green to orange-red (Fig. 4a, b). Similar to the PBBr copolymer, the RL characteristics of these copolymers are consistent with their PL spectra but with a larger proportion of phosphorescence (Supplementary Figs. 43–44 and 48–49, and Supplementary Tables 16–18). The main emission bands of these copolymers displayed long lifetimes of 10.68, 7.39, and 6.21 ms for PNBr, PDBr, and PIBr films, respectively, suggesting the phosphorescent radioluminescence feature under X-ray irradiation (Fig. 4c). Meanwhile, these copolymers also showed an obvious dependent relationship between radioluminescence intensity and the relative concentration of bromine atoms-containing monomers. As the content of bromine atoms was increased, the radioluminescence intensity of copolymers showed a significant increase (Supplementary Fig. 50a–c). Moreover, these resulting copolymers also presented good responsivity to different X-ray dose rates and photostability under a high dose rate of X-ray (278 μGy s^−1^) excitation (Supplementary Figs. 50–53).

### Potential applications of the phosphorescent polymeric scintillators

**Fig. 5:**
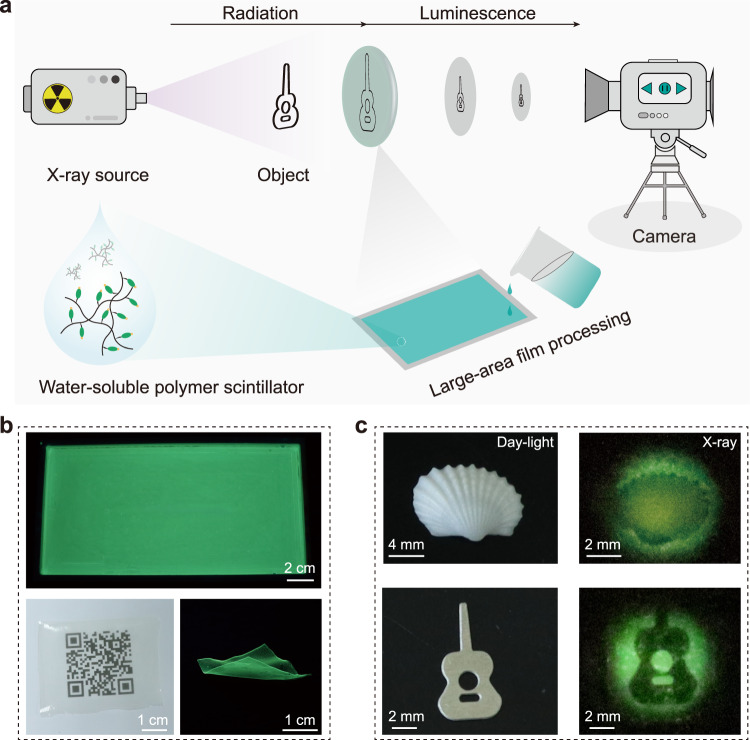
Demonstration of the flexible phosphorescent copolymers for potential applications in digital radiography. **a** A schematic of the radiography set-up, and the preparation process of X-ray imaging background substrate using PNBr-10 aqueous solution. **b** Photographs of large-scale, transparent, and flexible PNBr-10 copolymer film under daylight and UV light, respectively. **c** X-ray images of the shell and guitar-shaped sheet metal under X-ray irradiation.

Given the processibility and X-ray responsive radioluminescence features of the resulting copolymers, we explored their potential applications in X-ray radiography. As shown in Fig. 5a, we employ the copolymer PNBr film as the intrinsically scintillating material for detecting the radiography of imaged object. Benefiting from the water solubility and good film-forming ability of copolymer PNBr, we fabricate a transparent, flexible, and uniform polymer film with a large scale of 20 × 10 cm, which displayed bright yellow-green emission and could be formed in a bendable shape for potential applications in flexible display and imaging as shown in Fig. 5b and Supplementary Fig. 54. Then, the shell and metal sheet were placed between the X-ray source and the polymer film. Under X-ray radiation, the photography of a small-sized shell and a guitar could be clearly visualized using a Canon camera from the polymer film, respectively (Fig. 5c). These results demonstrate the potential of amorphous copolymers as large-area fabricated X-ray detectors in multiple application scenarios.

## Discussion

In conclusion, we have presented a facile strategy to realize X-ray-excited phosphorescent scintillation from amorphous copolymers through simple radical binary copolymerization of the bromine-substituted chromophores and acrylic acid. We found that both the absorption of X-rays and the efficiency of the phosphorescent materials play a crucial role in obtaining efficient radioluminescence. The introduction of the heavy bromine atoms on the organic chromophores not only promotes X-ray absorption but also facilitates the ISC process, which is beneficial for obtaining polymeric scintillators with good X-ray-responsibility and photostability. Meanwhile, it was found that the content of the bromine atom-containing monomers is also important to optimize the radioluminescence performance of the copolymers. Excitedly, based on this design principle, we obtained a series of copolymers featuring multi-color radioluminescence ranging from green to orange-red, and demonstrated the potential of amorphous copolymers for X-ray radiography. Our findings not only reveal the X-ray-excited phosphorescence properties of copolymers with good processibility, transparency, and flexibility, but also demonstrate the potential applications of organic phosphorescent polymers for large-area fabricated X-ray imaging.

## Methods

### Preparation of monomers

*4-(allyloxy)-4*′*-bromo-1,1*′*-biphenyl (BBr)*: 4-Bromo-4′-hydroxybiphenyl (1.00 g; 4.01 mmol), KOH (0.56 g; 10.03 mmol), Allyl bromide (0.58 g; 4.81 mmol) were dissolved in 30 mL *N,N*-dimethylformamide. After the solution was stirred for 5 h at 40 °C, the solvent was removed by rotary evaporation and the residue was purified by column chromatography to give BBr (0.85 g, 73.28%) as a white solid. ^1^H NMR (500 MHz, CDCl_3_) δ 7.63–7.37 (m, 6H), 7.08–6.94 (m, 2H), 6.11 (m, 1H), 5.48 (dd, 1H), 5.35 (dd, 1H), 4.61 (dt, 2H). ^13^C NMR (126 MHz, CDCl_3_) δ 158.45 (s), 139.73 (s), 133.18 (s), 132.65 (s), 131.82 (s), 128.32 (s), 127.98 (s), 120.83 (s), 117.84 (s), 115.17 (s), 68.91 (s).

*4-(allyloxy)-1,1*′*-biphenyl (BPh)*: 4-Phenylphenol (1.00 g; 5.88 mmol), KOH (0.82 g; 14.70 mmol), Allyl bromide (0.85 g; 7.06 mmol) were dissolved in 30 mL *N,N*-dimethylformamide. After the solution was stirred for 5 h at 40 °C, the solvent was removed by rotary evaporation and the residue was purified by column chromatography to give BPh (0.9 g, 72.6%) as a white solid. ^1^H NMR (500 MHz, CDCl_3_) δ 7.66–7.54 (m, 4H), 7.47 (t, 2H), 7.40–7.31 (m, 1H), 7.11–6.94 (m, 2H), 6.22–6.06 (m, 1H), 5.50 (dd, 1H), 5.36 (dd, 1H), 4.63 (d, 2H). ^13^C NMR (126 MHz, CDCl_3_) δ 158.21 (s), 140.84 (s), 133.96 (s), 133.32 (s), 128.76 (s), 128.17 (s), 126.75 (d, J = 7.6 Hz), 117.77 (s), 115.07 (s), 68.93 (s).

*2-(allyloxy)-6-bromonaphthalene (NBr)*: Following the similar synthetic procedure as BBr, the reaction of 6-bromo-2-naphthol (1.00 g, 4.48 mmol), KOH (0.63 g; 11.20 mmol), Allyl bromide (0.65 g; 5.38 mmol), in *N,N*-dimethylformamide 30 mL for 5 h yielded NBr as a white solid (0.95 g, 81.20%). ^1^H NMR (500 MHz, CDCl_3_) δ 7.95 (dd, 1H), 7.65 (dd, 1H), 7.60 (t, 1H), 7.56–7.44 (m, 1H), 7.32–7.25 (m, 1H), 7.26–7.16 (m, 1H), 6.21–6.09 (m, 1H), 5.55–5.42 (m, 1H), 5.42–5.29 (m, 1H), 4.68 (dt, 2H). ^13^C NMR (126 MHz, CDCl_3_) δ 156.84 (s), 132.99 (d), 130.09 (s), 129.65 (d), 128.55 (s), 128.42 (s), 120.04 (s), 117.98 (s), 117.14 (s), 107.01 (s), 68.89 (s).

*2,2*′*-bis(allyloxy)-6,6*′*-dibromo-1,1*′*-binaphthalene (DBr)*: Following the similar synthetic procedure as BBr, the reaction of 6,6′-Dibromo-1,1′-bi-2-naphthol (1.00 g, 2.25 mmol), KOH (0.63 g; 11.25 mmol), Allyl bromide (0.68 g; 5.63 mmol), in *N,N*-dimethylformamide 30 mL for 5 h yielded DBr as a faint yellow solid (0.76 g, 64.40%). ^1^H NMR (500 MHz, CDCl_3_) δ 8.04 (d, 2H), 7.86 (d, 2H), 7.44 (d, 2H), 7.30 (dd, 2H), 7.02 (d, 2H), 5.82–5.72 (m, 2H), 5.09–4.99 (m, 4H), 4.55 (dt, 4H). ^13^C NMR (126 MHz, CDCl_3_) δ 154.27 (s), 133.34 (s), 132.52 (s), 130.32 (s), 129.87 (s), 129.66 (s), 128.51 (s), 127.11 (s), 119.85 (s), 117.48 (s), 116.71 (s), 116.43 (s), 69.82 (s).

*2-allyl-6-bromo-1H-benzo[de]isoquinoline-1,3(2H)-dione (IBr)*: 4-Bromo-1,8-naphthalic anhydride (1.00 g; 3.61 mmol), NH_3_·H_2_O (0.19 g; 5.41 mmol), Allyl bromide (0.65 g; 5.41 mmol) were dissolved in 50 mL ethanol. After the solution was stirred for 5 h at 60 °C, the solvent was removed by rotary evaporation and the residue was purified by column chromatography to give IBr (0.35 g, 30.70%) as a yellow solid. ^1^H NMR (500 MHz, CDCl_3_) δ 8.64 (d, 1H), 8.55 (d, 1H), 8.39 (d, 1H), 8.04 (d, 1H), 7.89–7.80 (m, 1H), 6.01 (dt, 1H), 5.35 (dd, 1H), 5.25 (dd, 1H), 4.81 (d, 2H). ^13^C NMR (126 MHz, CDCl_3_) δ 163.27 (d), 133.33 (s), 132.13 (s), 131.94 (s), 131.31 (s), 131.11 (s), 130.60 (s), 130.36 (s), 128.98 (s), 128.08 (s), 122.98 (s), 122.11 (s), 117.88 (s), 42.53 (s).

### Preparation of polymers

*PBBr*: Take the PBBr-800 as an example. The polymer was synthesized by radical copolymerization. BBr (0.025 g, 0.086 mmol), acrylic acid (4.98 g, 69.16 mmol), 2,2′-azobis(2-methylpropionitrile) (AIBN) (0.025 g) were dissolved in toluene 60 mL under nitrogen atmosphere. After this solution was stirred at 80 °C for 18 h, the mixture was cooled to room temperature and the white solids were obtained by filtration. Then, the crude product was washed with dichloromethane three times, which was dissolved in deionized water and dialyzed by a dialysis tube (MWCO = 2000) for 72 h. The solution was kept at 60 °C for 12 h, finally, the transparent polymer film was obtained.

*PBPh*: Following the same synthetic procedure as PBBr, the reaction of BPh (0.9 g, 4.28 mmol), acrylic acid (3.08 g, 42.80 mmol), AIBN (0.025 g), in toluene solution 50 mL for 18 h yielded PBPh-10 as a transparent film.

*PNBr*: Following the same synthetic procedure as PBBr, the reaction of NBr (0.020 g, 0.086 mmol), acrylic acid (4.95 g, 68.67 mmol), AIBN (0.025 g), in toluene solution 50 mL for 18 h yielded PNBr-800 as a transparent film.

*PDBr*: Following the same synthetic procedure as PBBr, the reaction of DBr (0.045 g, 0.076 mmol), acrylic acid (4.38 g, 60.81 mmol), AIBN (0.025 g), in toluene solution 60 mL for 18 h yielded PDBr-800 as a transparent film.

*PIBr*: Following the same synthetic procedure as PBBr, the reaction of IBr (0.030 g, 0.095 mmol), acrylic acid (5.47 g, 75.91 mmol), AIBN (0.025 g), in toluene solution 70 mL for 18 h yielded PIBr-800 as a transparent film.

*PAA*: The polymer was synthesized by radical copolymerization. Acrylic acid (5 g, 69.39 mmol), 2,2′-azobis(2-methylpropionitrile) (AIBN) (0.025 g) were dissolved in toluene 70 mL under nitrogen atmosphere. After this solution was stirred at 80 °C for 18 h, the mixture was cooled to room temperature and the white solids were obtained by filtration. Then, the crude product was washed with dichloromethane three times, which was dissolved in deionized water and dialyzed by a dialysis tube (MWCO = 2000) for 72 h. The solution was kept at 60 °C for 12 h, finally, the transparent polymer film was obtained.

## Supplementary information

Supplementary Information

Description of Additional Supplementary Files

Supplementary Movie 1

## Data Availability

The authors declare that the data supporting the findings of this study are available in the paper and its supplementary information files, or available from the corresponding authors on reasonable request. Source data are provided with this paper.

## Source data

Source Data
