# Supplementary material for: Organic phosphorescent scintillation from copolymers by X-ray irradiation
Source: Nat Commun. 2022 Jul 9;13:3995. doi: 10.1038/s41467-022-31554-3 (PMC9271082; doi:10.1038/s41467-022-31554-3)
Supplement: Supplementary file 1 — Supplementary Information [file 41467_2022_31554_MOESM1_ESM.pdf]

Supplementary Information

Organic phosphorescent scintillation from copolymers by X-ray irradiation

Nan Gan[1†], Xin Zou[1†], Mengyang Dong[1], Yanze Wang[1], Xiao Wang[1], Anqi Lv[2], Zhicheng Song[2], Yuanyuan Zhang[1], Wenqi Gong[2], Zhu Zhao[2], Ziyang Wang[1], Zixing Zhou[2], Huili Ma[2], Xiaowang Liu[1], Qiushui Chen[3], Huifang Shi[2], Huanghao Yang[3], Long Gu[1*], Zhongfu An[2*], Wei Huang[1,2*]

[1]Frontiers Science Center for Flexible Electronics (FSCFE), MIIT Key Laboratory of Flexible Electronics (KLoFE), Northwestern Polytechnical University, Xi'an 710072, P.R. China.

[2]Key Laboratory of Flexible Electronics (KLoFE) & Institute of Advanced Materials (IAM), Nanjing Tech University (NanjingTech), 30 South Puzhu Road, Nanjing, 211816, P.R. China.

[3]MOE Key Laboratory for Analytical Science of Food Safety and Biology, State Key Laboratory of Photocatalysis on Energy and Environment, College of Chemistry, Fuzhou University, Fuzhou, 350108, P.R. China.

[†]These authors contributed equally to this work

**Correspondence to: iamlgu@nwpu.edu.cn; iamzfan@njtech.edu.cn; iamwhuang@njtech.edu.cn*

Supplementary Methods

Reagents and materials

Unless otherwise stated, raw materials used in the experiments were purchased from commercial sources without further purification unless special instructions. AIBN was recrystallized twice in ethanol. For flash column chromatography, silica gel with 200 ~ 300 mesh was used.

Measurements

Nuclear magnetic resonance (^{1}H and ^{13}C NMR) spectra were recorded on a Bruker 500 MHz spectrometer using D_2O, $CDCl_3$ or DMSO as the solvent. Chemical shifts were referred to the internal standard tetramethylsilane (TMS). Resonance patterns were reported with the notation s (singlet), d (doublet), t (triplet), q (quartet), and m (multiplet). Aqueous gel permeation chromatography (GPC) measurements were performed on WYATT DAWN (SEC-MALS) detected by an LS laser detector. Samples used 0.1 mol/L $NaNO_3$ solution as mobile phase at 1.0 mL/min flow rate. Steady-state fluorescence and phosphorescence spectra, and lifetimes were measured using a fluorescence spectrophotometer (Edinburgh FLS1000) equipped with a xenon arc lamp (Xe900), a nanosecond hydrogen flash-lamp (nF920), or a microsecond flash-lamp (μF900), and all the phosphorescence spectra were recorded with a delay time of 8 ms. Photoluminescence efficiencies were collected on a Hamamatsu absolute PL quantum yield spectrometer C11347. TEM photographs and EDS spectra were obtained on a FEI Talos F200X TEM. Wide-angle X-ray scattering (WAXS) patterns were achieved from a Xenocs Nanoinxider with Cu-Kα micro source (40 mm) at 30 W. Powder X-ray diffraction patterns were recorded on a Bruker D8 Advance X-ray diffractometer with Cu-Kα radiation. X-ray excited luminescent spectra were recorded using an Edinburgh FS5 fluorescence spectrophotometer (Edinburgh Instruments Ltd.) with a miniature X-ray source (AMPEK, Inc.). Luminescent photos were taken by a Canon EOS 850D camera.

Computational details

The excitation energies for the singlet and triplet states were obtained by the TD-DFT (density functional theory) method at Pysoc/B3LYP/DEF$_2$-SVP level, and all the DFT calculations were performed using Gaussian 09 program (Revision C. 01).[1]

Differences between photoluminescence and radioluminescence

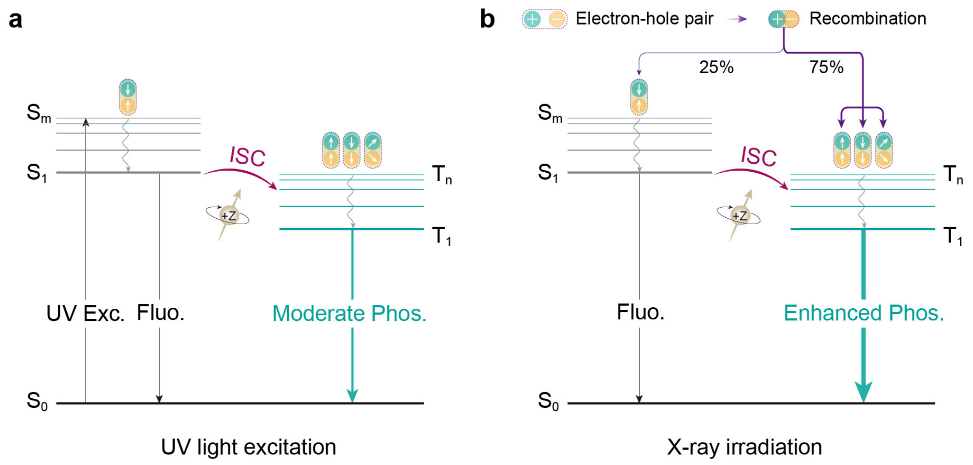

Supplementary Figure 1. A comparison of luminescence mechanism between (**a**) UV light excitation and (**b**) X-ray irradiation.

According to the mechanism of phosphorescent radioluminescence, the absorption of X-ray and the efficiency of the materials play a crucial role in obtaining phosphorescence radioluminescence under X-ray irradiation. The X-ray absorptivity of materials can determine the generation of excitons after the recombination of holes and electrons. For one scintillator, atomic number *Z* can largely influence the X-ray stopping power (attenuation coefficient μ), which can be presented as:

$$\mu = \frac{\rho Z^4}{AE^3} \propto Z^4 \quad (1)$$

where ρ denotes the material density, A represents the atomic mass, and E is the X-ray photon energy.

As shown in Supplementary Figure 1, the differences between radioluminescence and photoluminescence lies in the starting absorption stage rather than the decay stage. For UV light excitation, the molecules are excited from the ground state to high-level singlet states, and then the triplet excitons can only be generated through the ISC process from singlet excitons. The relationship between phosphorescence efficiency (Φ_p), fluorescence efficiency (Φ_f), rate constant of fluorescent radiation (k_f), $S_1 \rightarrow S_0$ internal conversion (k_{ic}), phosphorescent radiation (k_p), and non-radiative decay rate of T_1 (k_{nr}) can be expressed as:

$$\Phi_{f,UV} = \frac{k_f}{k_f + k_{ic} + k_{isc}} \quad (2)$$

$$\Phi_{isc} = \frac{k_{isc}}{k_f + k_{ic} + k_{isc}} \quad (3)$$

$$\Phi_{p,UV} = \Phi_{isc} \frac{k_p}{k_p + k_{nr}} \quad (4)$$

In the situation of X-ray irradiation, massive electrons and holes are first produced after the X-ray source excites the inner electrons of heavy atoms. Then, the electrons and holes recombine to directly produce singlet and triplet excitons at an approximate ratio of 1:3, according to spin-statistics. The radiative decay processes of singlet and triplet excitons generate fluorescence and phosphorescence, respectively. Importantly, in addition to the recombination of holes and electrons, the triplet excitons can also generate from singlet excitons that

produced by recombination through intersystem crossing. Hence, an additional channel emerged for the population of triplet excitons under X-ray irradiation than UV excitation. The fluorescence and phosphorescence quantum yields of materials can be written as below:

$$\Phi_{\mathrm{f,X-ray}} = \delta \cdot \mathrm{x} \frac{k_{\mathrm{f}}}{k_{\mathrm{f}} + k_{\mathrm{ic}} + k_{\mathrm{isc}}} \quad (5)$$

$$\Phi_{\mathrm{p,X-ray}} = \delta \cdot (1 - \mathrm{x} + \mathrm{x} \cdot \Phi_{\mathrm{isc}}) \frac{k_{\mathrm{p}}}{k_{\mathrm{p}} + k_{\mathrm{nr}}} \quad (6)$$

Here, δ is the number of electron-hole pairs generated by one X-ray photon, x is the proportion of singlet excitons by recombination. It is worth noting that photoluminescence and radioluminescence have the same decay processes of excited states, including the radiative and non-radiative pathways. These decay rate constants are determined by the intrinsic properties of molecules, including energy levels, molecular orbital types, etc.

General procedure for the synthesis of monomers and copolymers

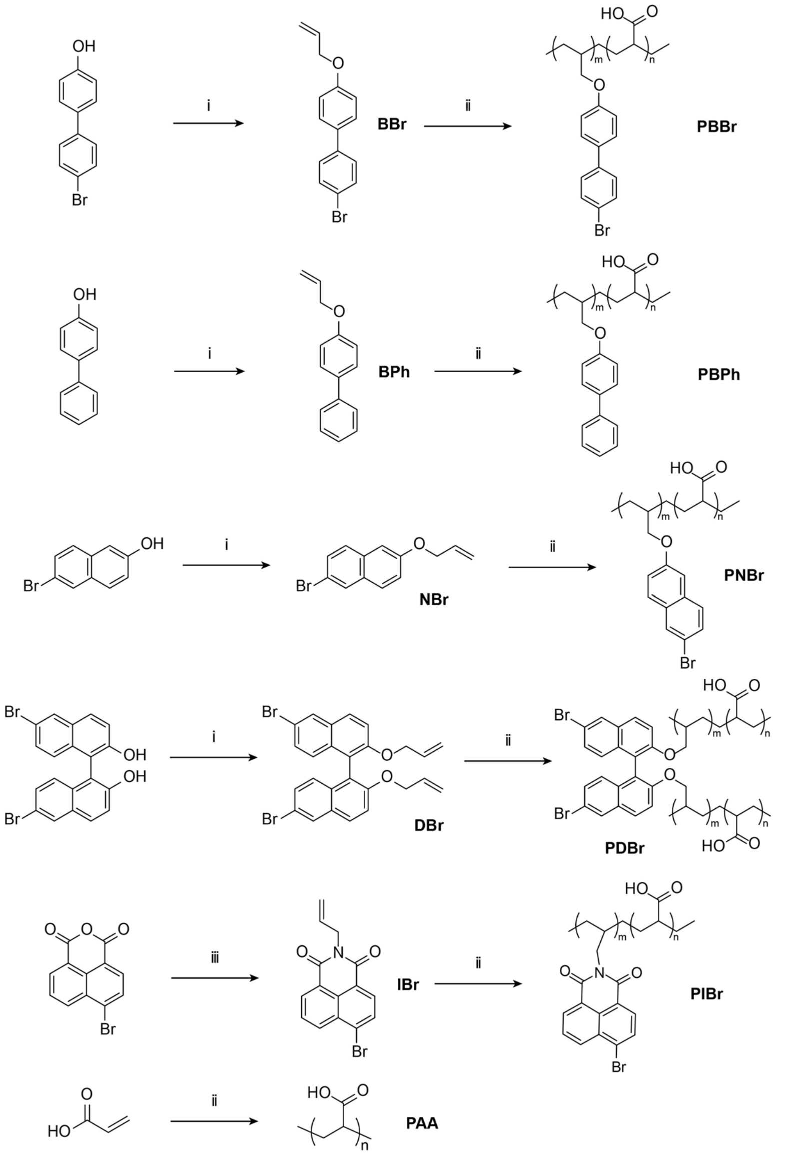

Supplementary Figure 2. Synthetic routes of binary copolymers. (i) KOH, DMF, Allyl bromide, 40°C, 5 h; (ii) Acrylic acid. AIBN, toluene, 80°C, 18 h. (iii) $NH_3 \cdot H_2O$, Allyl bromide, ethyl alcohol, 60°C, 4 h.

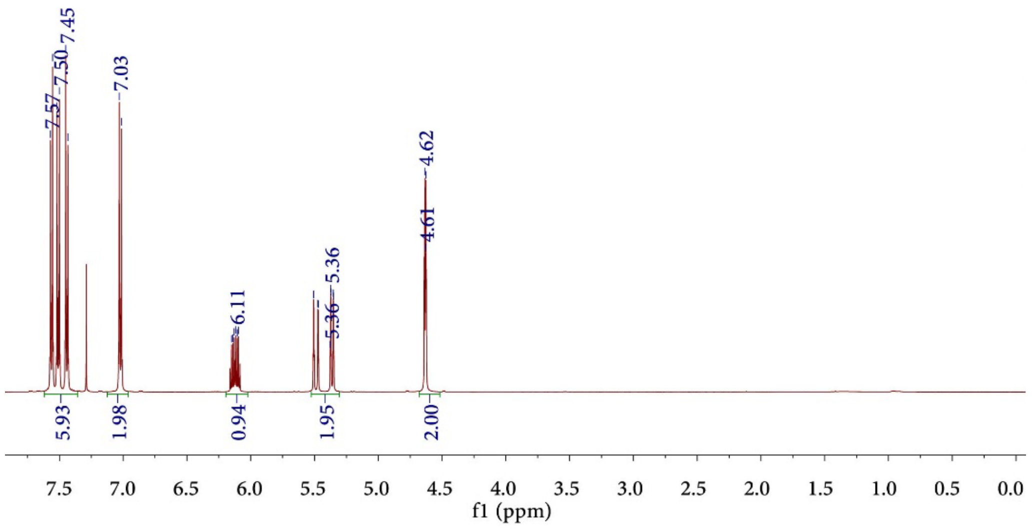

Supplementary Figure 3. ^{1}H NMR spectrum of BBr monomer in $CDCl_3$.

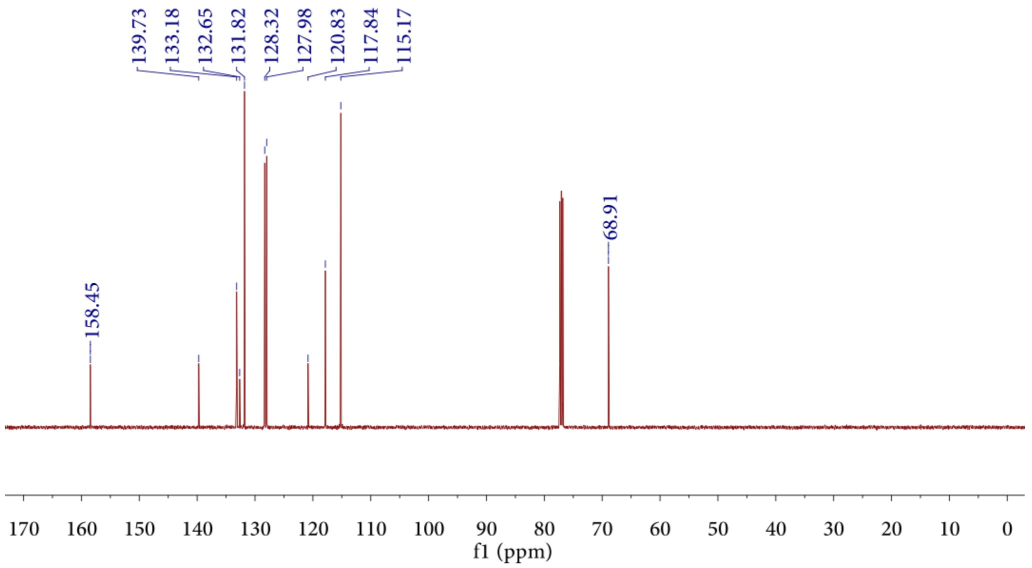

Supplementary Figure 4. ^{13}C NMR spectrum of BBr monomer in $CDCl_3$.

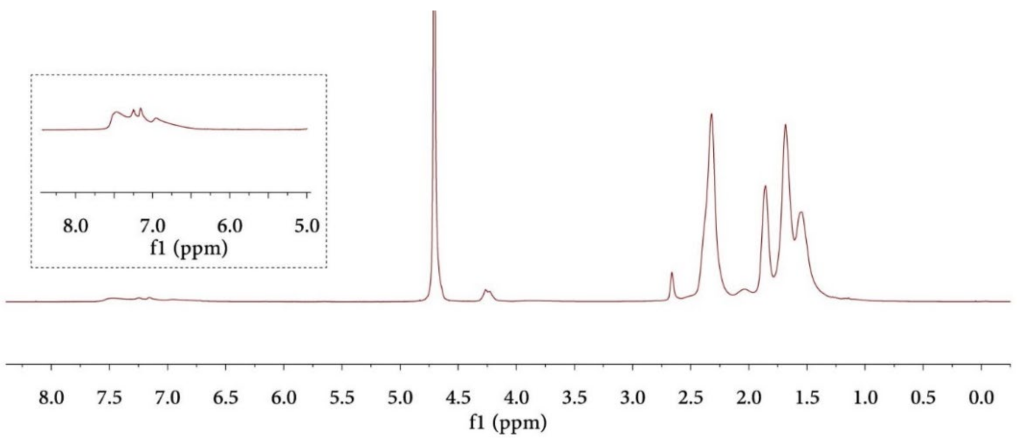

Supplementary Figure 5. ^{1}H NMR spectrum of polymer PBBr in D_2O.

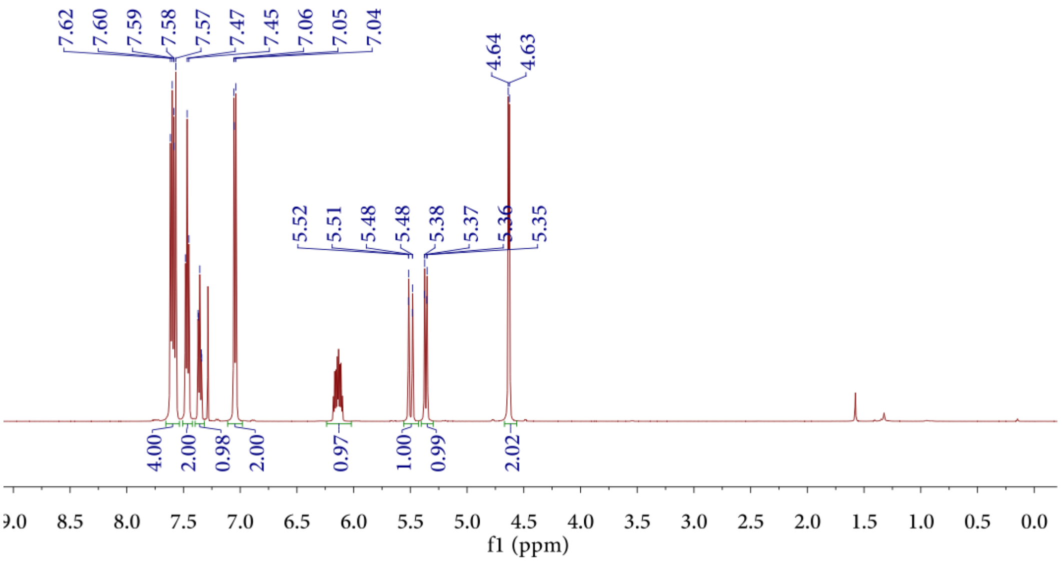

Supplementary Figure 6. ^{1}H NMR spectrum of BPh monomer in $CDCl_3$.

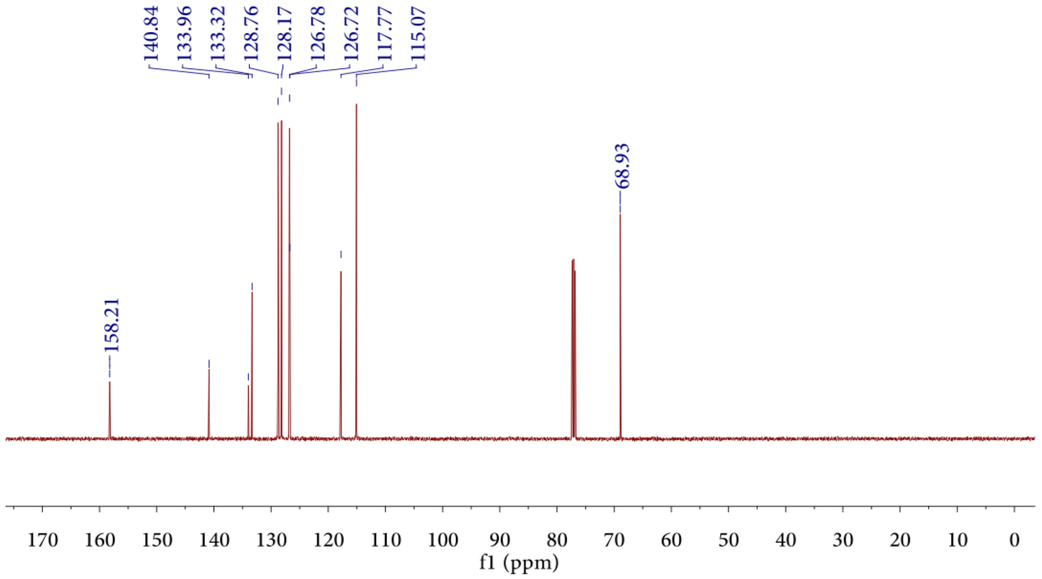

Supplementary Figure 7. ^{13}C NMR spectrum of BPh monomer in $CDCl_3$.

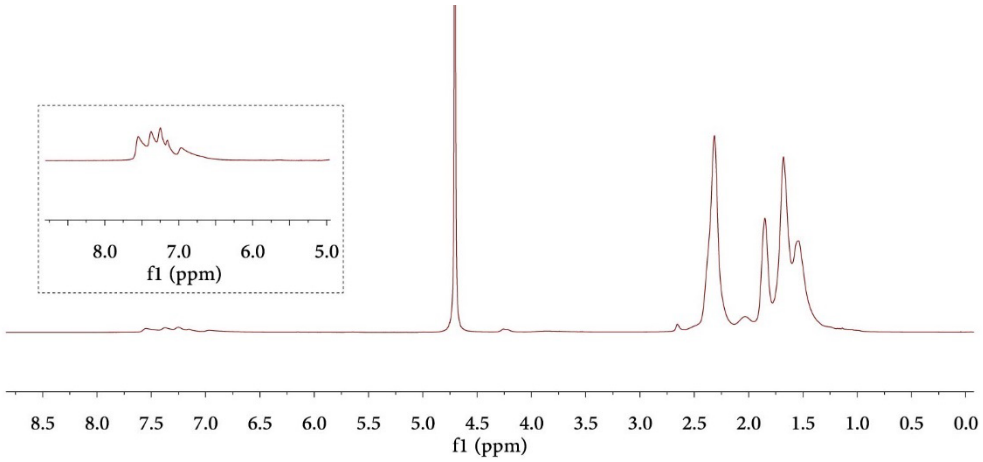

Supplementary Figure 8. ^{1}H NMR spectrum of polymer PBPh in D_2O.

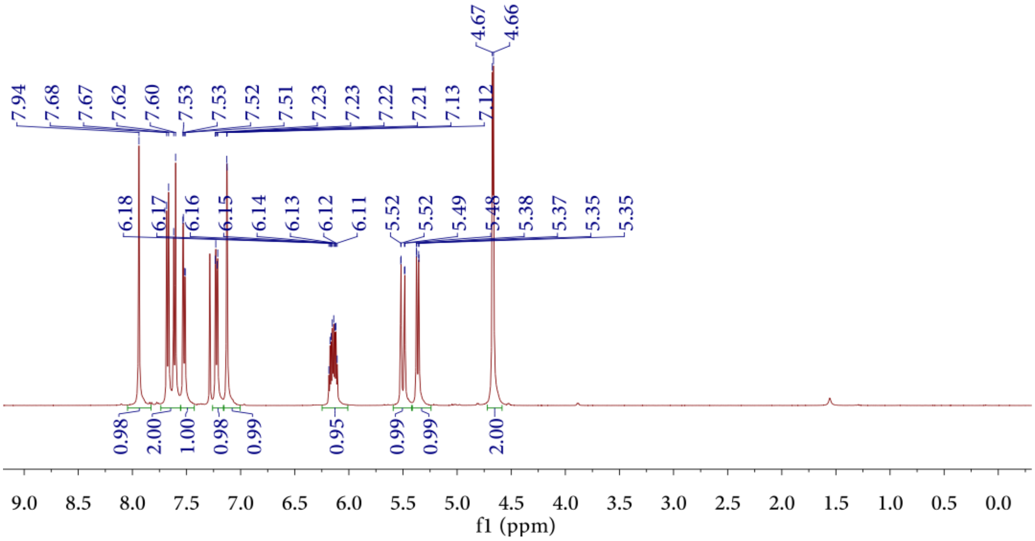

Supplementary Figure 9. ^{1}H NMR spectrum of NBr monomer in $CDCl_3$.

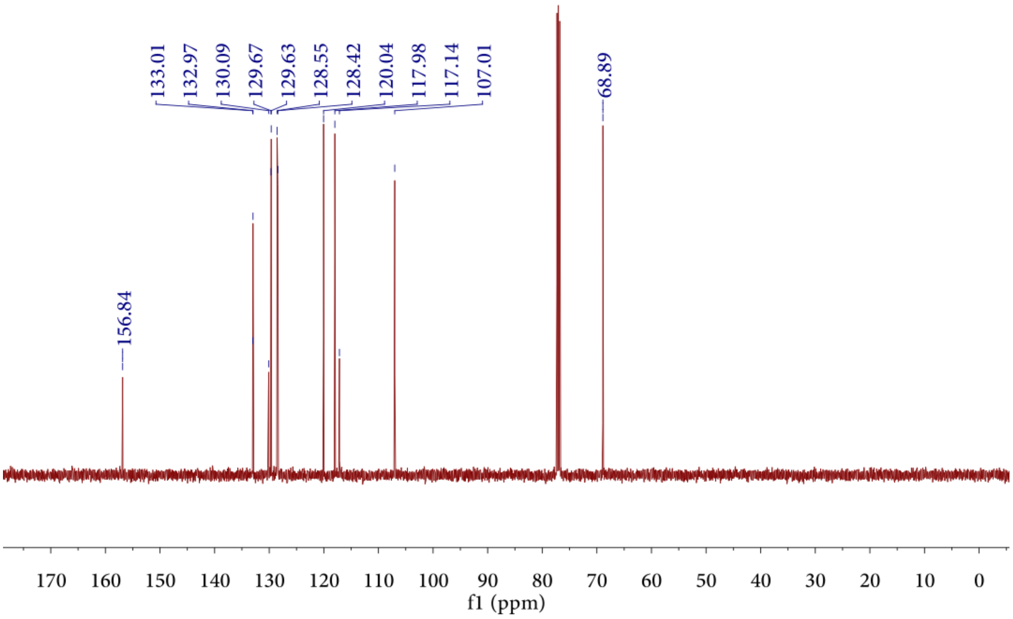

Supplementary Figure 10. ^{13}C NMR spectrum of NBr monomer in $CDCl_3$.

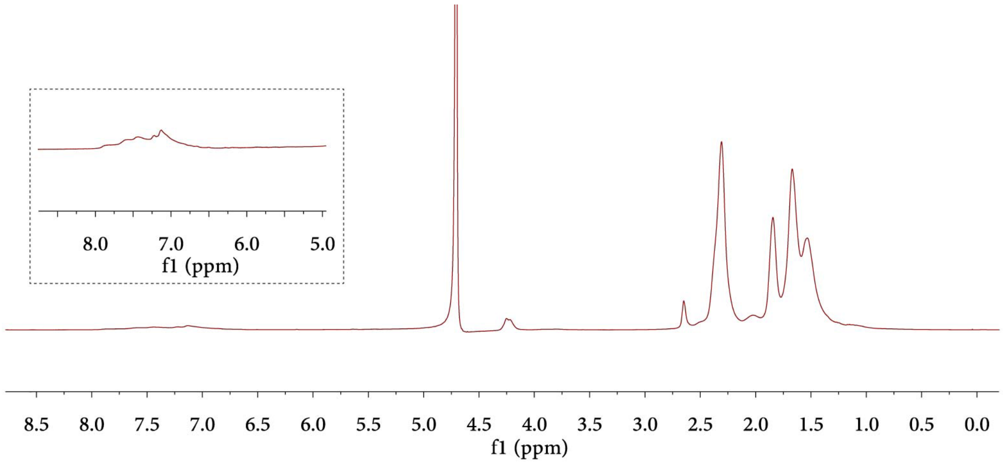

Supplementary Figure 11. ^{1}H NMR spectrum of polymer PNBr in D_2O.

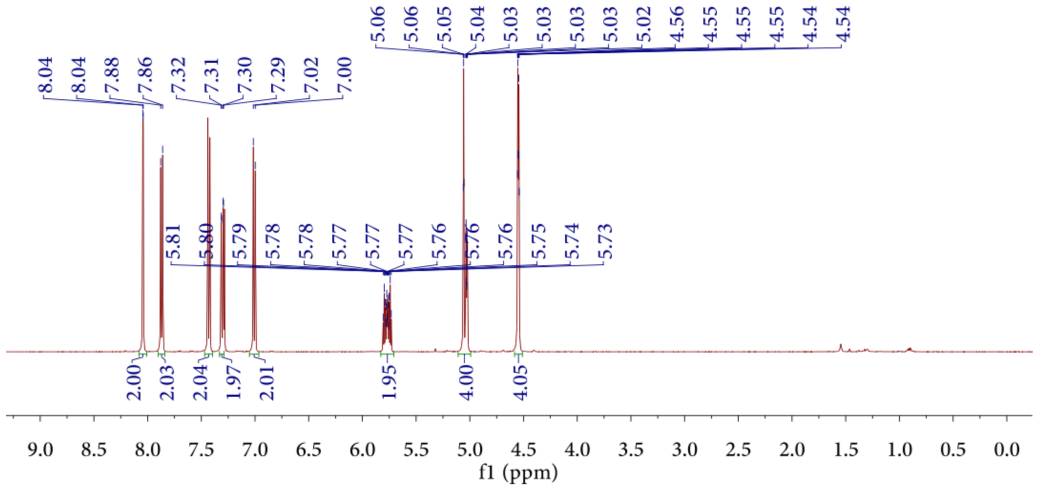

Supplementary Figure 12. ^{1}H NMR spectrum of DBr monomer in $CDCl_3$.

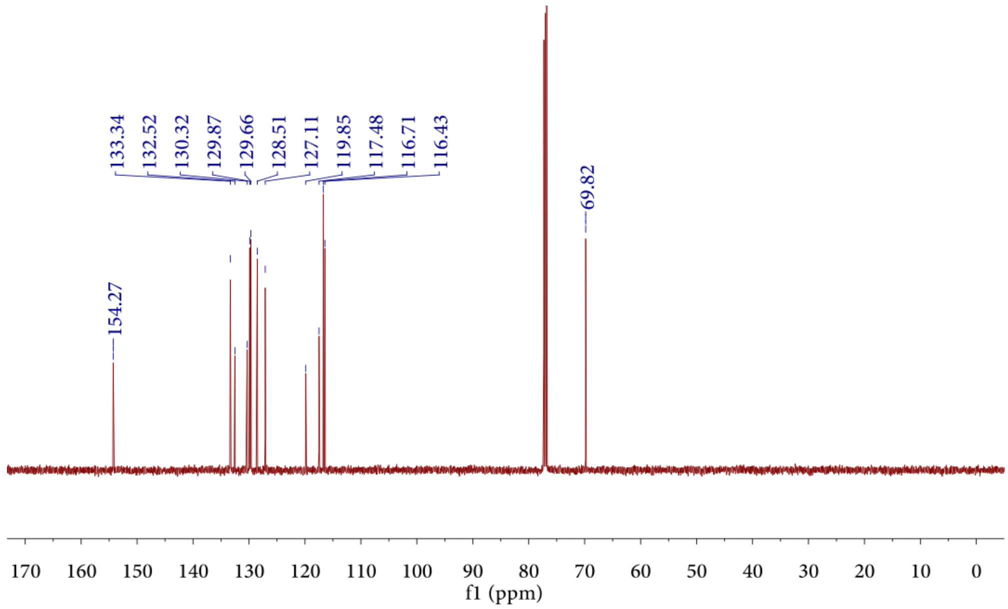

Supplementary Figure 13. ^{13}C NMR spectrum of DBr monomer in $CDCl_3$.

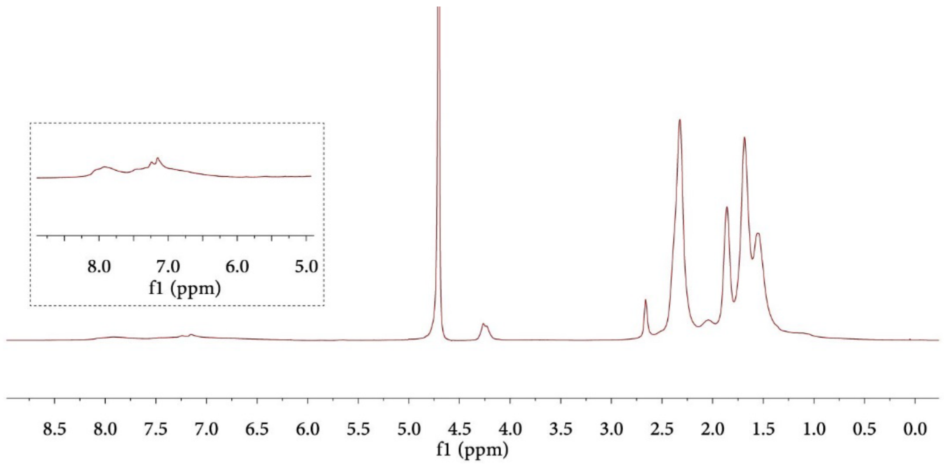

Supplementary Figure 14. ^{1}H NMR spectrum of polymer PDBr in D_2O.

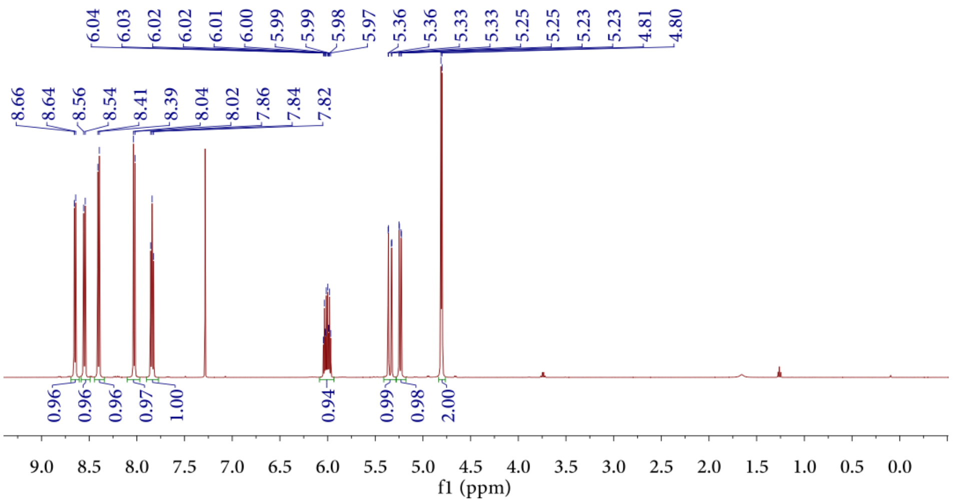

Supplementary Figure 15. ^1H NMR spectrum of IBr monomer in $CDCl_3$.

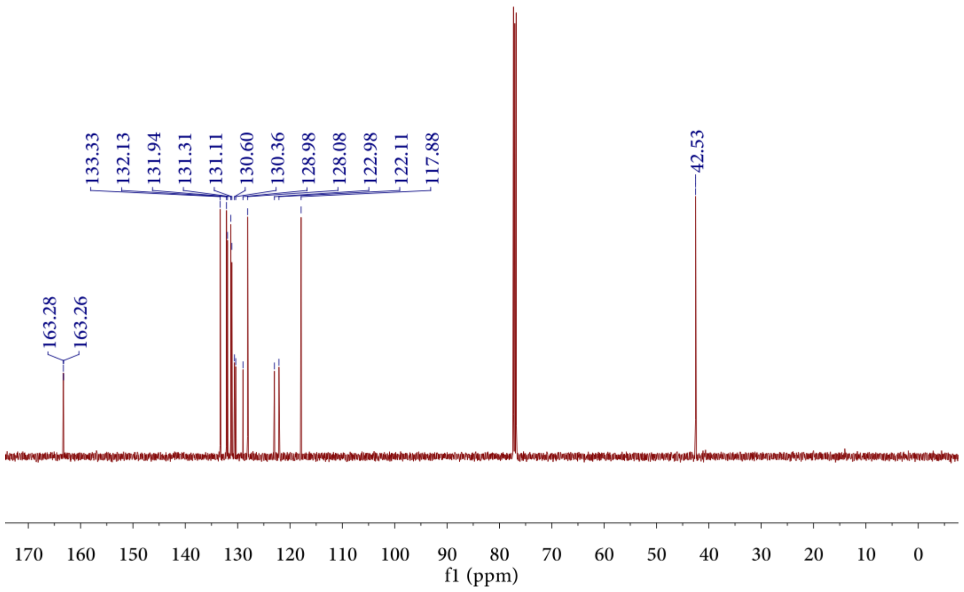

Supplementary Figure 16. ^{13}C NMR spectrum of IBr monomer in $CDCl_3$.

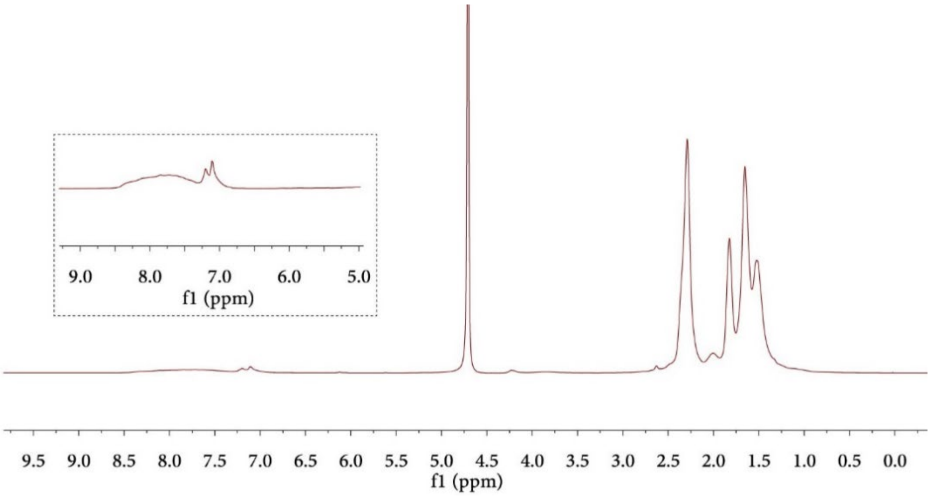

Supplementary Figure 17. ^1H NMR spectrum of polymer PIBr in D_2O.

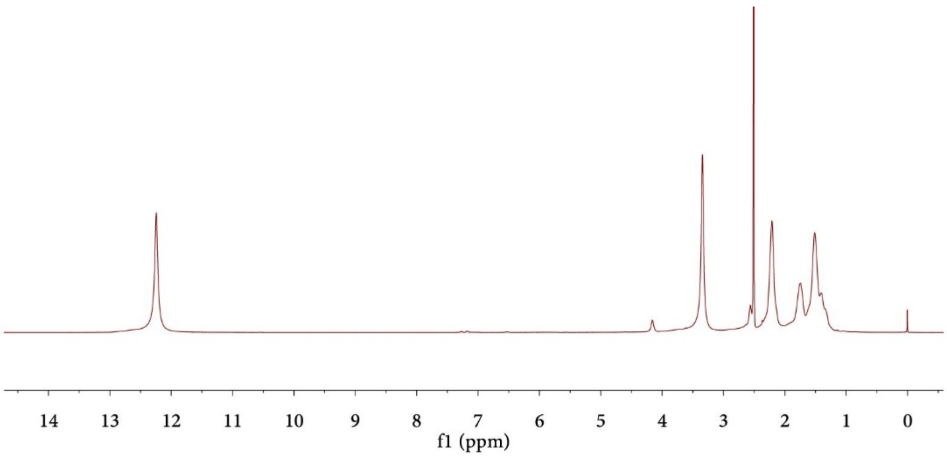

Supplementary Figure 18. ^1H NMR spectrum of polymer PAA in DMSO-d_6.

Supplementary Table 1. Aqueous gel permeation chromatography characterizations of copolymers.

Sample	PBBr-50	PNBr-50	PDBr-50	PIBr-50
Mw (Da)	35980	42740	145500	39200
Mn (Da)	32790	35440	69390	27920
PDI	1.097	1.206	2.097	1.404

The transmission electron microscopes (TEM) demonstrate the uniform and transparent film structures of PBBr copolymers. Elementary mapping images of the various polymers reveal the distribution of C, H, O, and Br elements, and Br atoms were well distributed in copolymers. Meanwhile, energy-dispersive spectroscopy (EDS) spectrum analyses on the different elements suggest that the contents of Br gradually increased with the molar ratios of BBr/AA ranging from 1/800 to 1/3. Note that the bromine content is too low for PBBr-200, PBBr-400 and PBBr-800 copolymer films to analyze accurately, thus the corresponding element analysis tables were not presented.

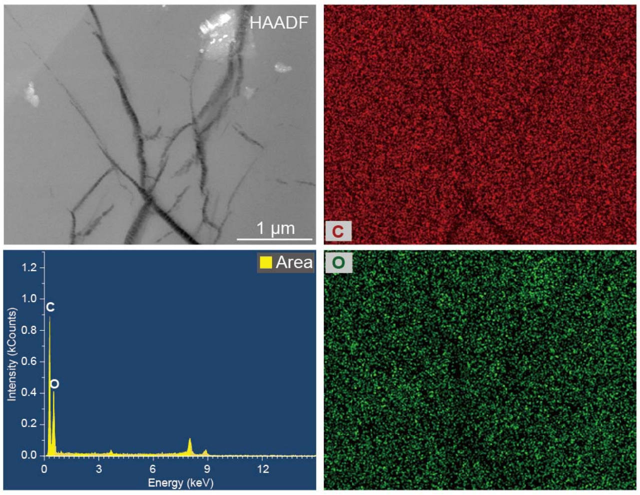

Supplementary Figure 19. Energy-dispersive spectroscopy spectrum and element mapping of PBPh-5 polymer showing the elemental distribution of C and O.

Supplementary Table 2. The corresponding element analysis of PBPh-5 polymer.

Element	Atomic Fraction (%)	Atomic Error (%)	Mass Fraction (%)	Mass Error (%)
C	75.9	2.48	70.2	1.51
O	24.1	1.27	29.8	1.64

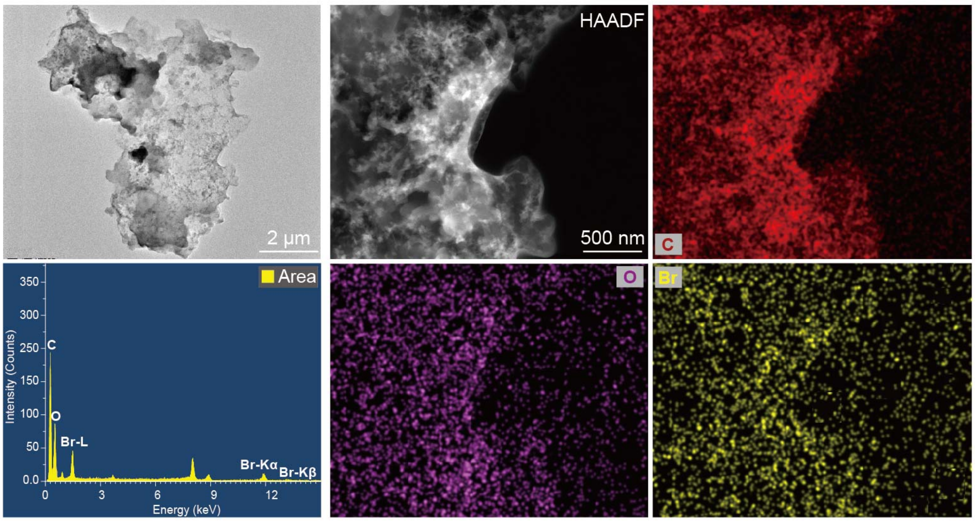

Supplementary Figure 20. TEM photograph, energy-dispersive spectroscopy spectrum, and element mapping of PBBr-3 polymer showing the elemental distribution of C, O, and Br, respectively.

Supplementary Table 3. The corresponding element analysis of PBBr-3 polymer.

Element	Atomic Fraction (%)	Atomic Error (%)	Mass Fraction (%)	Mass Error (%)
C	76.50	3.45	62.61	1.96
O	20.80	1.21	22.7	1.35
Br	2.70	0.38	14.69	2.10

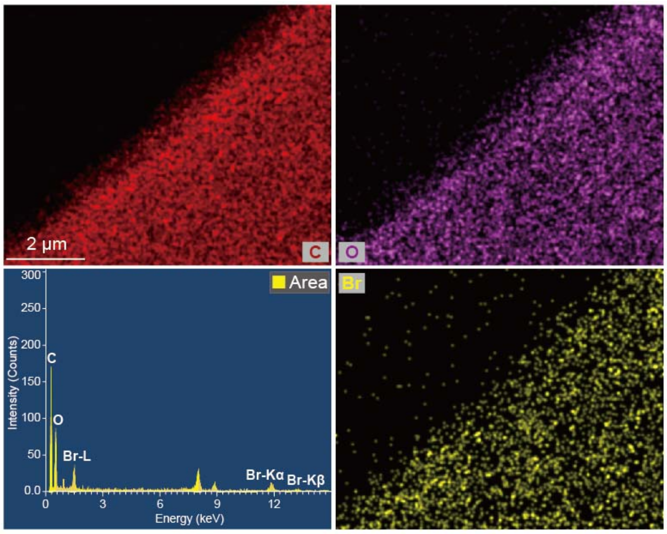

Supplementary Figure 21. Energy-dispersive spectroscopy spectrum, and element mapping of PBBr-5 polymer showing the elemental distribution of C, O, and Br, respectively.

Supplementary Table 4. The corresponding element analysis of PBBr-5 polymer.

Element	Atomic Fraction (%)	Atomic Error (%)	Mass Fraction (%)	Mass Error (%)
C	73.49	3.11	61.16	1.80
O	24.39	1.23	27.08	1.42
Br	2.12	0.29	11.76	1.64

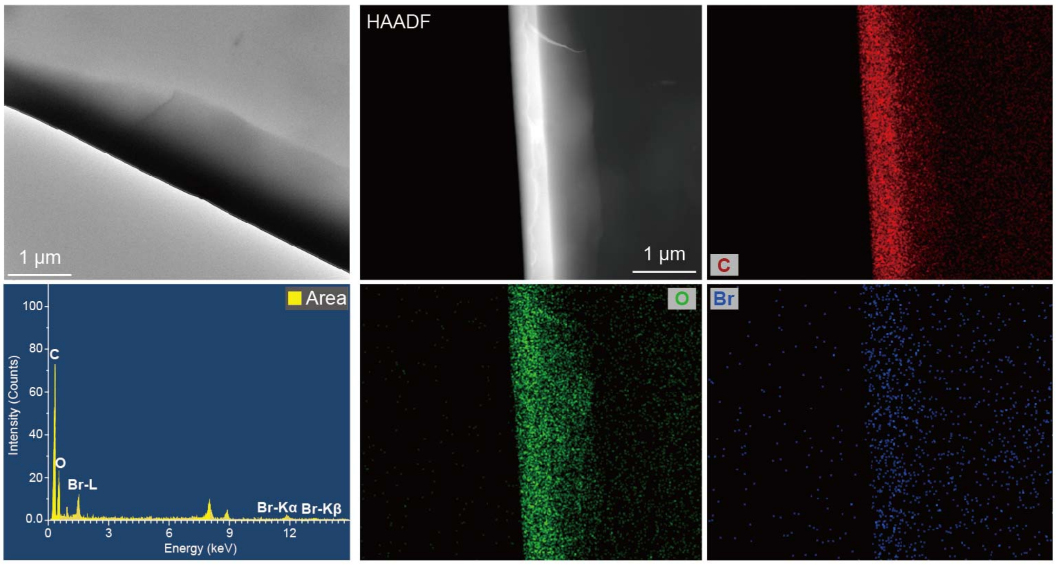

Supplementary Figure 22. TEM photograph, energy-dispersive spectroscopy spectrum, and element mapping of PBBr-10 polymer showing the elemental distribution of C, O, and Br, respectively.

Supplementary Table 5. The corresponding element analysis of PBBr-10 polymer.

Element	Atomic Fraction (%)	Atomic Error (%)	Mass Fraction (%)	Mass Error (%)
C	80.42	6.75	71.28	4.52
O	18.36	2.50	21.69	2.90
Br	1.22	0.65	7.03	3.87

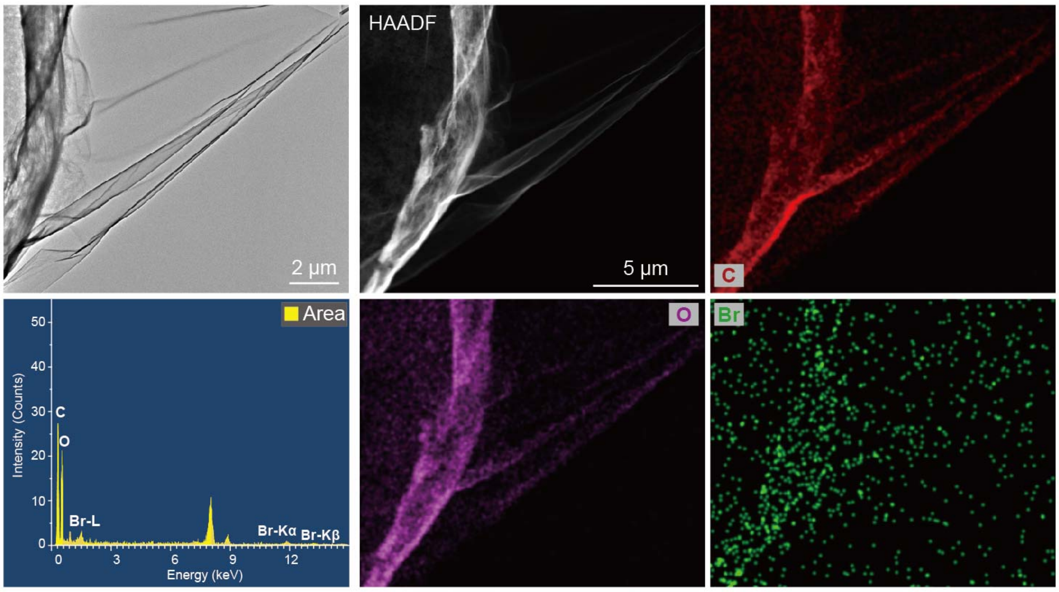

Supplementary Figure 23. TEM photograph, energy-dispersive spectroscopy spectrum, and element mapping of PBBr-20 polymer showing the elemental distribution of C, O, and Br, respectively.

Supplementary Table 6. The corresponding element analysis of PBBr-20 polymer.

Element	Atomic Fraction (%)	Atomic Error (%)	Mass Fraction (%)	Mass Error (%)
C	65.81	6.75	57.20	4.23
O	33.51	5.26	38.84	5.11
Br	0.68	0.25	3.96	1.46

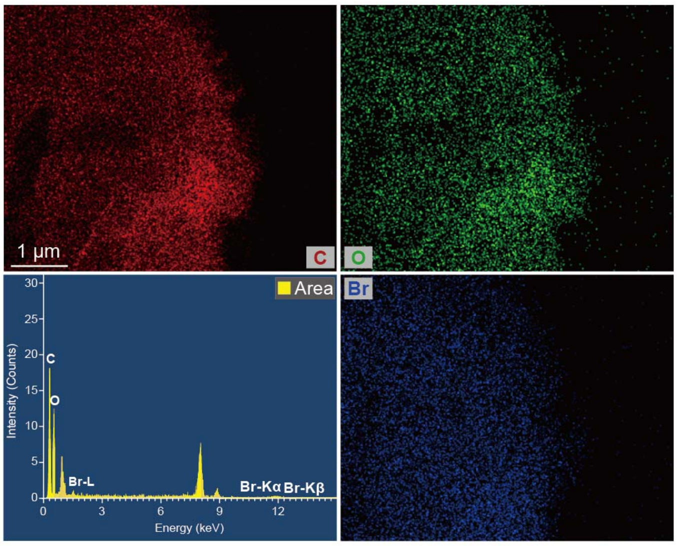

Supplementary Figure 24. Energy-dispersive spectroscopy spectrum and element mapping of PBBr-50 polymer showing the elemental distribution of C, O, and Br, respectively.

Supplementary Table 7. The corresponding element analysis of PBBr-50 polymer.

Element	Atomic Fraction (%)	Atomic Error (%)	Mass Fraction (%)	Mass Error (%)
C	63.30	9.05	55.76	4.86
O	36.45	8.96	42.77	9.33
Br	0.25	0.13	1.47	0.73

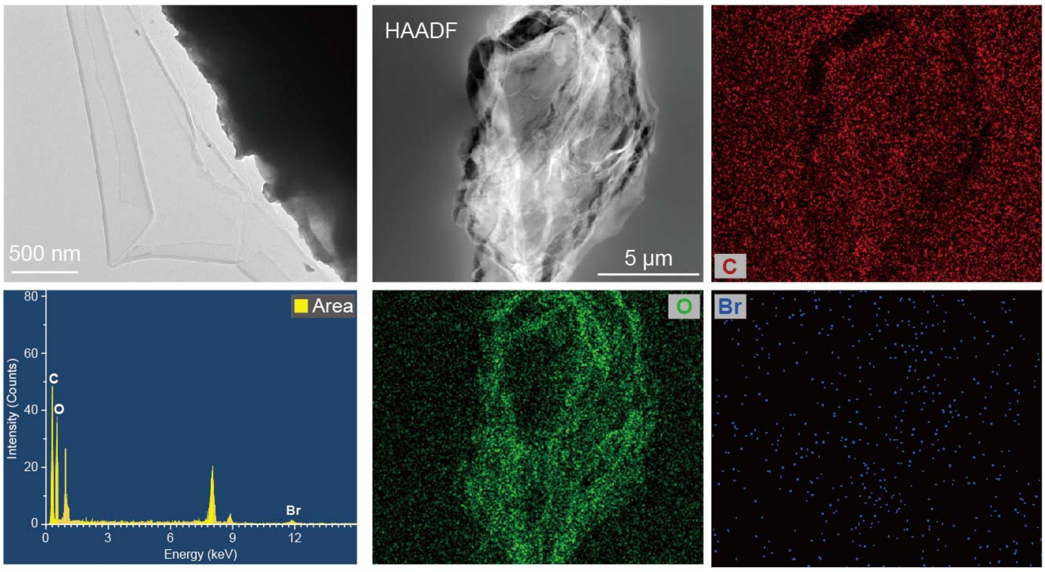

Supplementary Figure 25. TEM photograph, energy-dispersive spectroscopy spectrum, and element mapping of PBBr-100 polymer showing the elemental distribution of C, O, and Br, respectively.

Supplementary Table 8. The corresponding element analysis of PBBr-100 polymer.

Element	Atomic Fraction (%)	Atomic Error (%)	Mass Fraction (%)	Mass Error (%)
C	63.17	9.22	55.78	6.08
O	36.67	3.40	43.26	4.05
Br	0.16	0.44	0.96	2.79

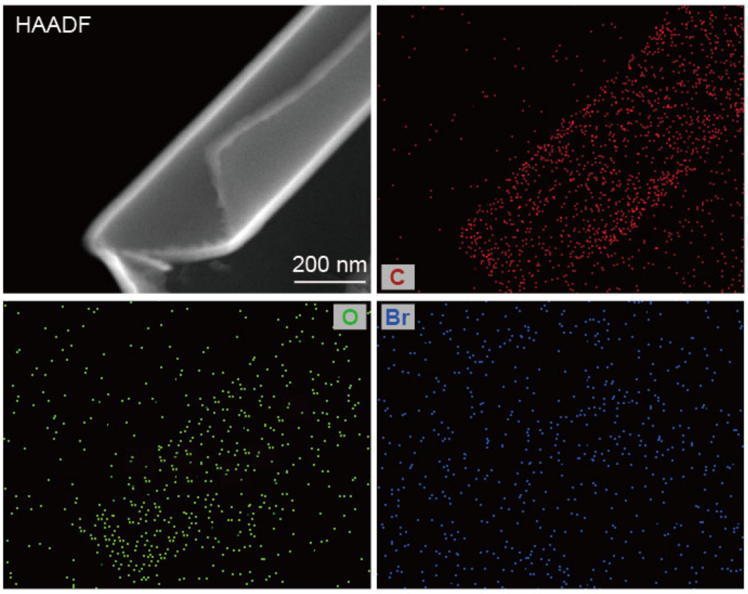

Supplementary Figure 26. Element mapping of PBBr-200 polymer showing the elemental distribution of C, O, and Br, respectively. Note: the bromine content is too low to analyze accurately.

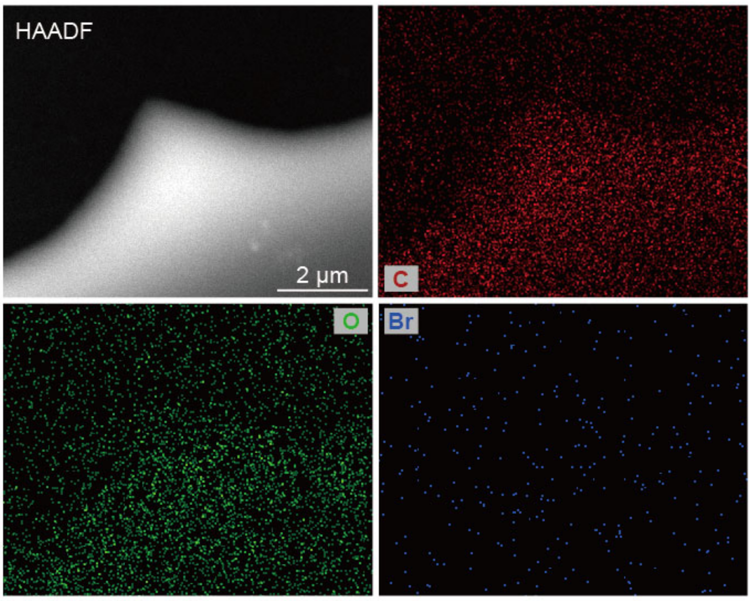

Supplementary Figure 27. Element mapping of PBBr-400 polymer showing the elemental distribution of C, O, and Br, respectively.

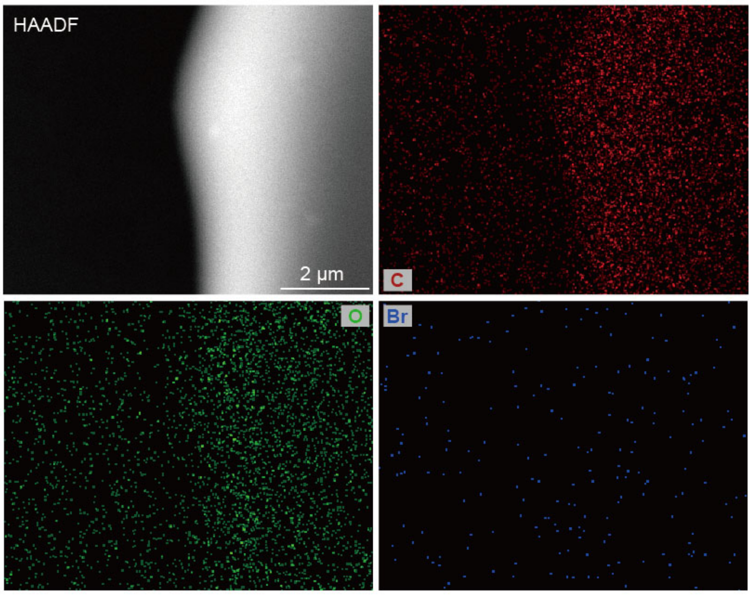

Supplementary Figure 28. Element mapping of PBBr-800 polymer showing the elemental distribution of C, O, and Br, respectively.

Additional photophysical properties of monomers and polymers in solution and film state.

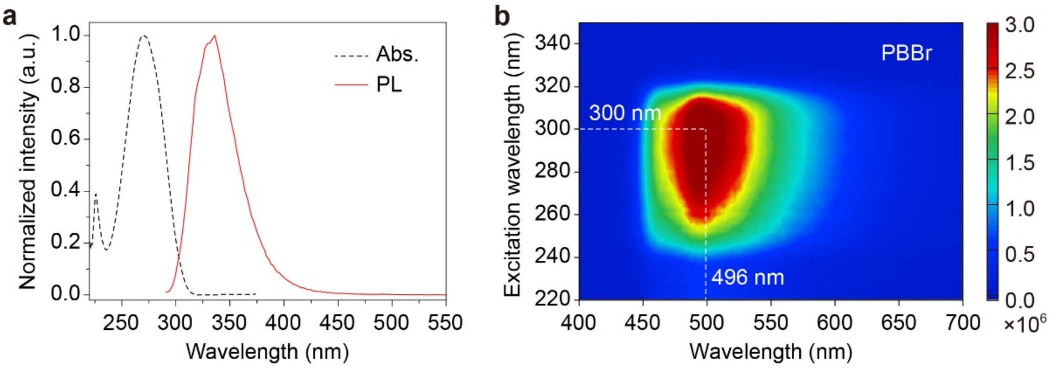

Supplementary Figure 29. **a** Absorption (black line) and photoluminescence (red line) spectra of BBr in dilute DCM solution (2×10^{-5} mol/L) excited by 270 nm under ambient conditions. **b** Excitation-phosphorescence emission mapping of PBBr-50 polymer film under ambient conditions.

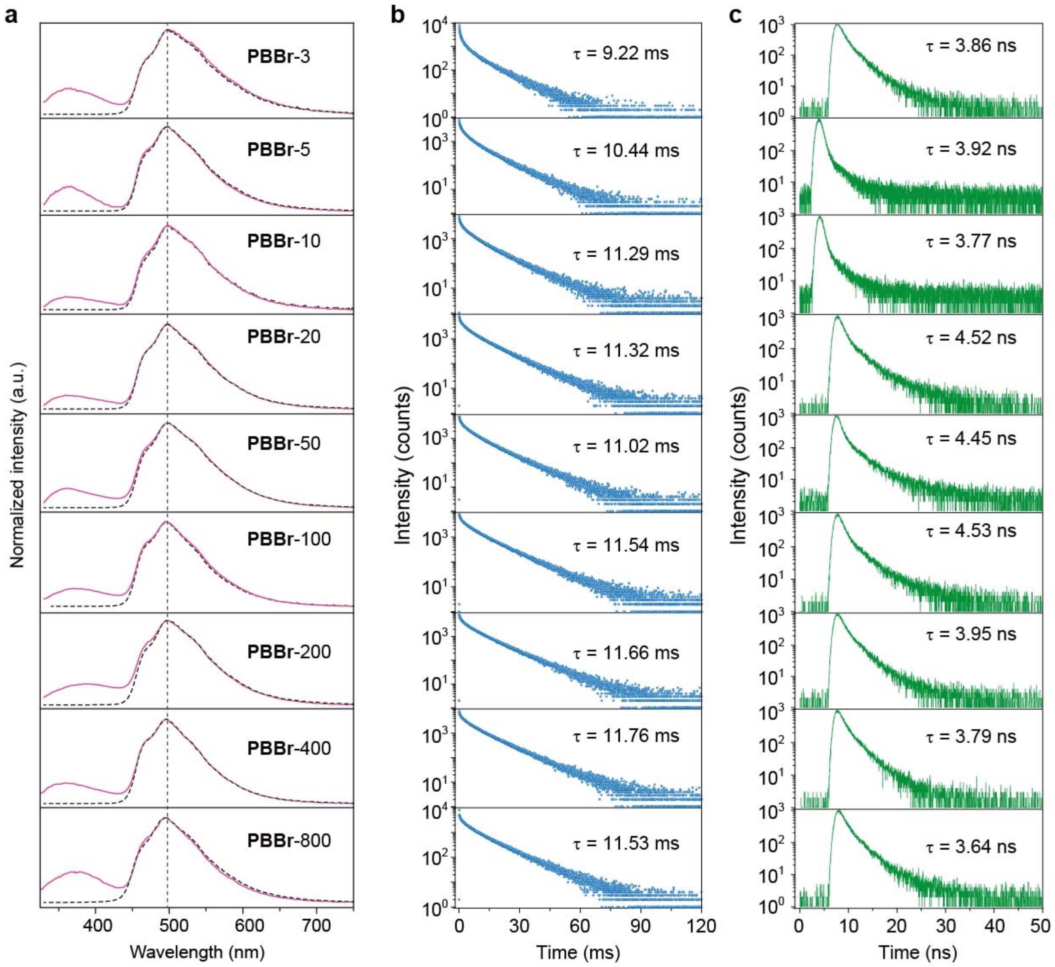

Supplementary Figure 30. **a**, **b**, and **c** Normalized steady-state photoluminescence (solid lines) and room temperature phosphorescence (dashed lines) spectra, and lifetime profiles of the emission bands at around 500 and 360 nm for PBBr-3 to PBBr-800 films, respectively.

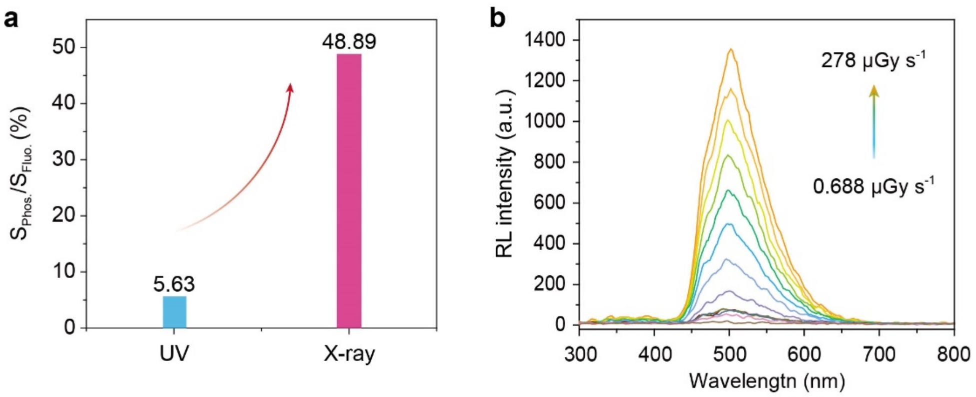

Supplementary Figure 31. **a** The ratios of $S_{Phos.}/S_{Fluo.}$ for PBBr-50 polymer film under the excitation of ultraviolet and X-rays, wherein the $S_{Phos.}$ and $S_{Fluo.}$ describe the integral area of room temperature phosphorescence and fluorescence region, respectively. **b** The dosage dependent emission of PBBr-5 film in the range of 0.688 to 278 μGy s^{-1}.

Supplementary Table 9. Luminescence lifetimes (τ) of various polymer films under ambient conditions.[a]

Polymer	Wavelength (nm)	Fluorescence				Phosphorescence			
		$τ_1$ (ns)	A_1 (%)	$τ_2$ (ns)	A_2 (%)	$τ_1$ (ms)	A_1 (%)	$τ_2$ (ms)	A_2 (%)
PBBr-3	365	0.59	77.21	3.86	22.79				
	497					1.61	28.35	9.22	71.65
PBBr-5	366	0.70	78.88	3.92	21.12				
	497	-	-	-	-	3.05	25.56	10.44	74.44
PBBr-10	376	0.65	74.88	3.77	25.12				
	498					3.95	23.90	11.29	76.10
PBBr-20	363	0.83	61.61	4.52	38.39				
	497					3.27	20.73	11.32	79.27
PBBr-50	360	0.98	57.87	4.45	42.13				
	498					3.17	21.28	11.02	78.72
PBBr-100	372	1.21	62.20	4.53	37.80				
	495					3.12	17.78	11.54	82.22
PBBr-200	379	1.45	61.30	3.95	38.70				
	497					3.34	17.46	11.66	82.54
PBBr-400	359	1.32	52.65	3.79	47.35				
	496					3.43	17.19	11.76	82.81
PBBr-800	375	1.25	44.39	3.64	55.61				
	498					2.90	17.65	11.53	82.35

[a] Determined from the fitting function of $I(t) = A_1e^{-t/τ1} + A_2e^{-t/τ2}$ according to the photoluminescence decay curves.

Supplementary Table 10. Photoluminescence and phosphorescence quantum efficiencies of PBBr polymer films with various molar ratios of monomers under exposure of UV light.

Polymer	PBBr-3	PBBr-5	PBBr-10	PBBr-20	PBBr-50	PBBr-100	PBBr-200	PBBr-400	PBBr-800
Φ_{PL} (%)	17.7	21.8	28.6	36.5	48.5	60.5	54.8	49.8	40.3
Φ_{Ph} (%)	14.3	17.9	25.5	33.5	40.7	51.4	44.6	41.1	31.5

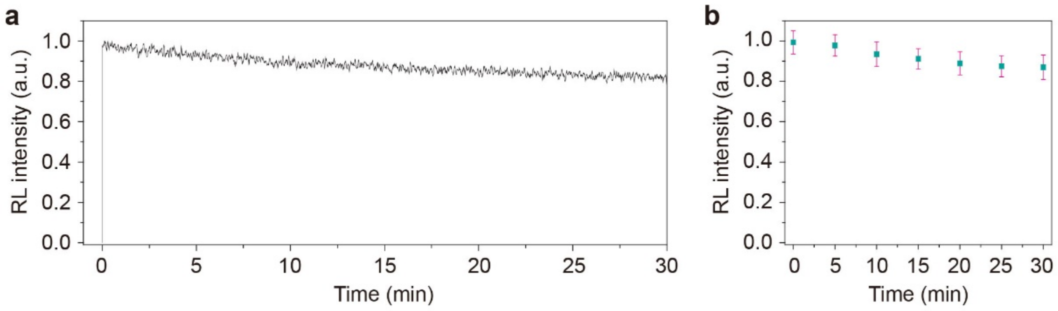

Supplementary Figure 32. **a** The radio-stability of the phosphorescence emission at 500 nm for PBBr-5 film under continuous X-ray irradiation for 30 min at a dose rate of 278 μGy s^{-1}. **b** Error bars were determined by three times repeated radio-stability measurements of the same PBBr film.

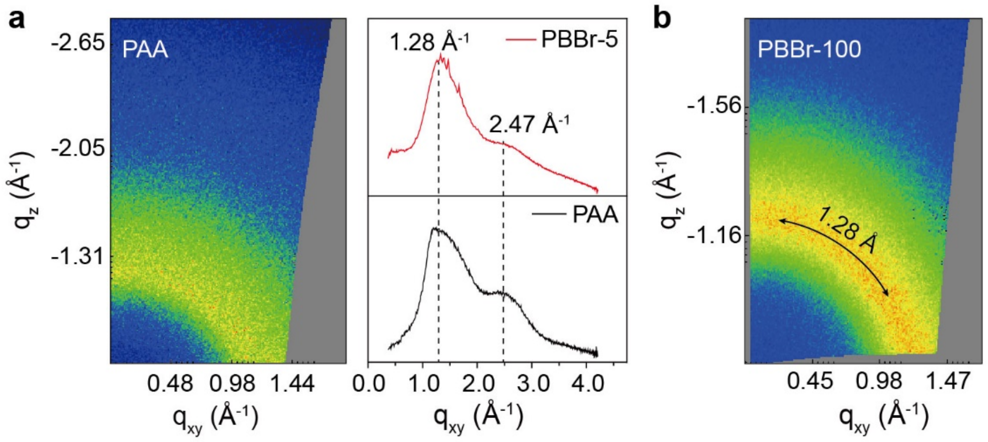

Supplementary Figure 33. Wide angle X-ray scattering patterns of polymer films (**a**) PAA[2] and (**b**) PBBr-100.

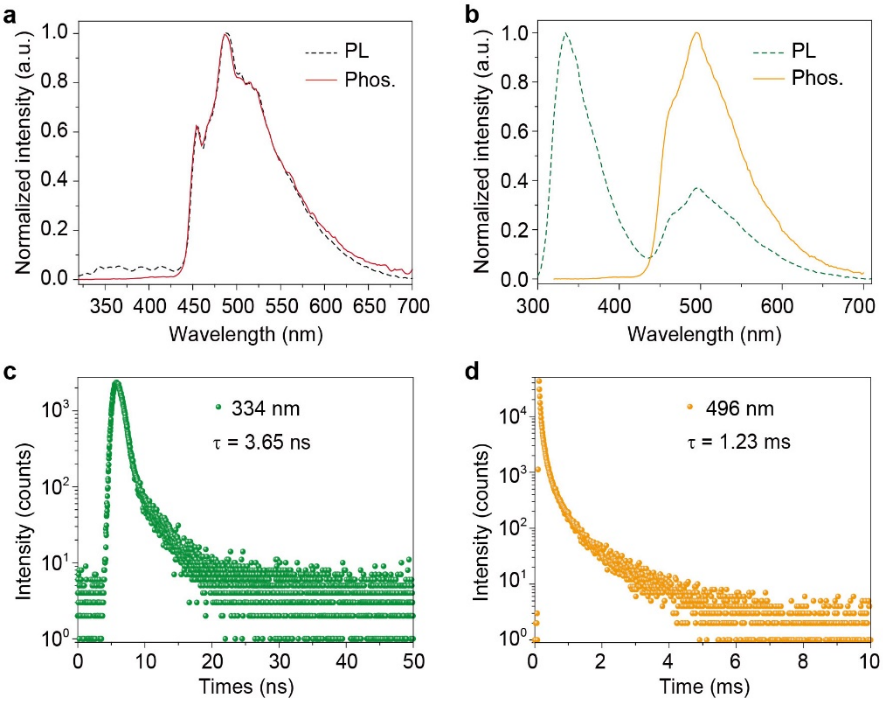

Supplementary Figure 34. **a** Steady-state PL (dashed line) and phosphorescence (solid line) spectra of BBr in dilute DCM solution (2×10^{-5} mol/L) excited at 280 nm at 77 K. **b** Steady-state PL (green line) and phosphorescence (yellow line) spectra of BBr doped in PMMA at 1 wt.% concentration excited by 280 nm under ambient conditions. **c**, and **d** Lifetime profiles of the PL emission band at 334 nm and phosphorescence emission peak at 496 nm for BBr doped in PMMA at 1 wt.% concentration under ambient conditions, respectively.

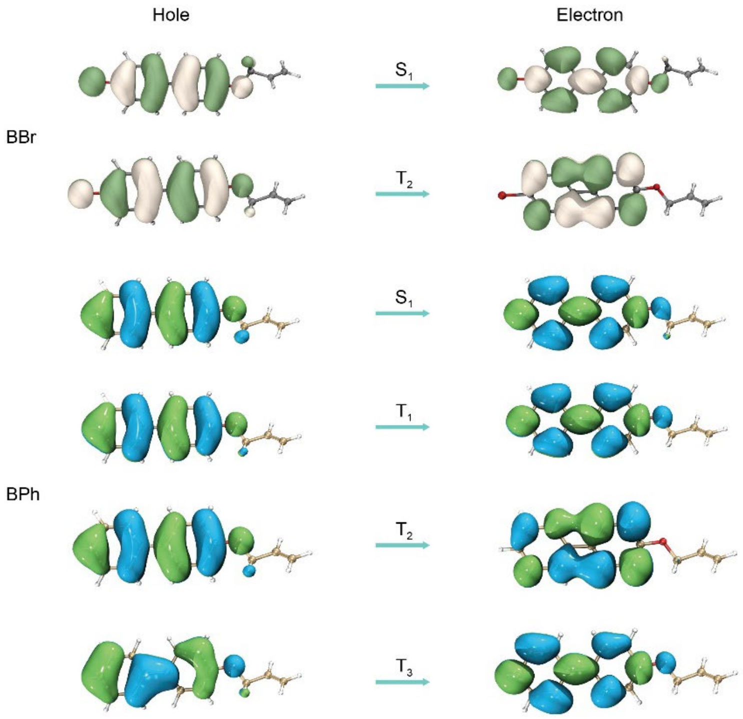

Supplementary Figure 35. Natural transition orbitals (NTOs) of monomers BBr and BPh using the TD-DFT method.

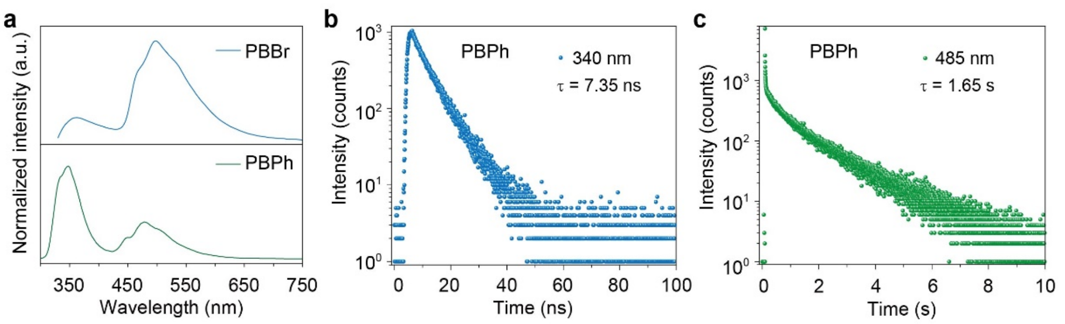

Supplementary Figure 36. **a** Normalized steady-state photoluminescence spectra of PBBr-5 and PBBh-5 polymer films under 290 nm UV light excitation. **b**, and **c** Lifetime profiles of the emission bands at 340 and 485 nm for PBPh films, respectively.

Supplementary Table 11. Luminescence lifetimes (τ) of PBPh-5 polymer film under ambient conditions.[a]

Polymer	Wavelength (nm)	Fluorescence				Phosphorescence					
		τ_1 (ns)	A_1 (%)	τ_2 (ns)	A_2 (%)	τ_1 (ms)	A_1 (%)	τ_2 (ms)	A_2 (%)	τ_3 (ms)	A_3 (%)
PBPh-5	340	3.43	23.24	7.35	76.76						
	485					45.58	1.45	455.1	26.25	1646.1	72.30

[a] Determined from the fitting function of $I(t) = A_1e^{-t/\tau_1} + A_2e^{-t/\tau_2}$ according to the photoluminescence decay curves.

Supplementary Table 12. Dynamic photophysical parameters of phosphorescent copolymers.

Polymer	τ_f (ns)	τ_p (ms)	Φ_f (%)	Φ_p (%)	k_{isc} (s^{-1}) [a]	k_p (s^{-1}) [b]	k_{nr} (s^{-1}) [c]
PBBr-5	3.92	10.44	3.9	17.9	2.45×10^8	17.8	78.0
PBPh-5	7.35	1646.1	9.41	5.29	1.23×10^8	0.035	0.57

The values are calculated according to the following formulas:[1]

$\Phi_{isc} = 1 - \Phi_f - \Phi_{ic} \approx 1 - \Phi_f$

$k_{isc} = \Phi_{isc} / \tau_f$ (Equation a)

$k_p = \Phi_p / (\Phi_{isc} \times \tau_p)$ (Equation b)

$k_{nr} = 1/\tau_p - k_p$ (Equation c)

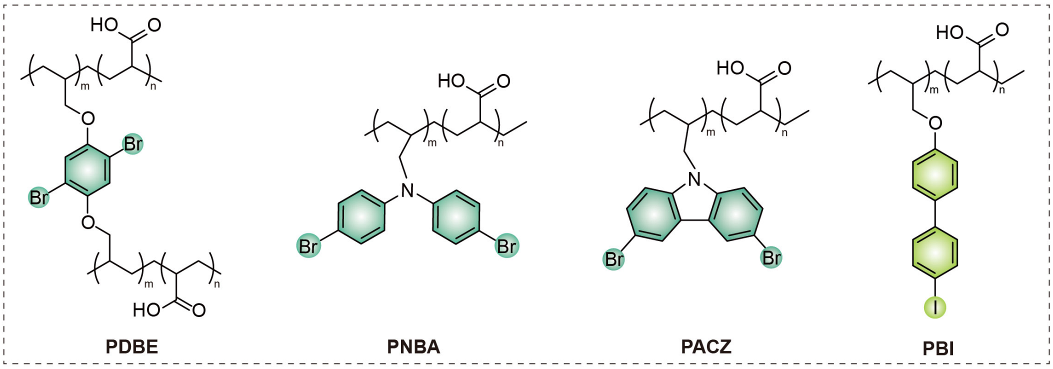

Supplementary Figure 37. Chemical structures of the copolymers PDBE, PNBA, PACZ and PBI.

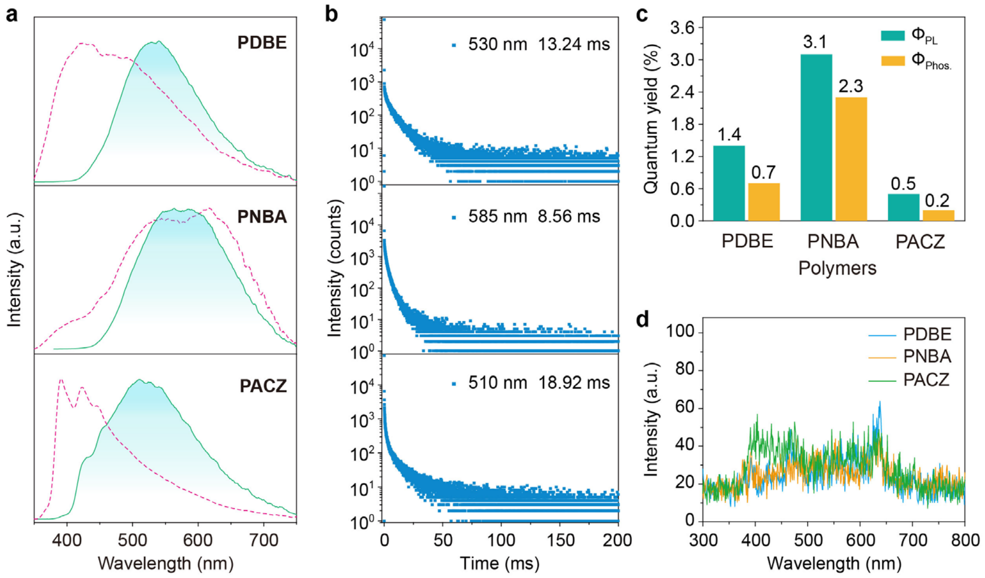

Supplementary Figure 38. **a** Normalized steady-state photoluminescence (red lines) and phosphorescence (green lines) spectra of the copolymers PDBE, PNBA, and PACZ under ambient conditions, respectively. **b** Lifetime profiles of the emission bands at 530, 585, and 510 nm for the copolymers PDBE, PNBA, and PACZ, respectively. **c** The histogram of PL and phosphorescence quantum yields. **d** RL spectra of the polymers PDBE, PNBA, and PACZ at a dose rate of 278 μGy s^{-1}.

Supplementary Table 13. Dynamic photophysical parameters of the phosphorescent copolymers.

Polymer	τ_f (ns)	τ_p (ms)	Φ_f (%)	Φ_p (%)	k_{isc} (s^{-1})	k_p (s^{-1})	k_{nr} (s^{-1})
PDBE	4.23	13.24	0.7	0.7	2.35×10^8	0.532	74.99
PNBA	5.01	8.56	0.8	2.3	1.98×10^8	2.71	114.1
PACZ	4.69	18.92	0.3	0.2	2.13×10^8	0.106	52.75

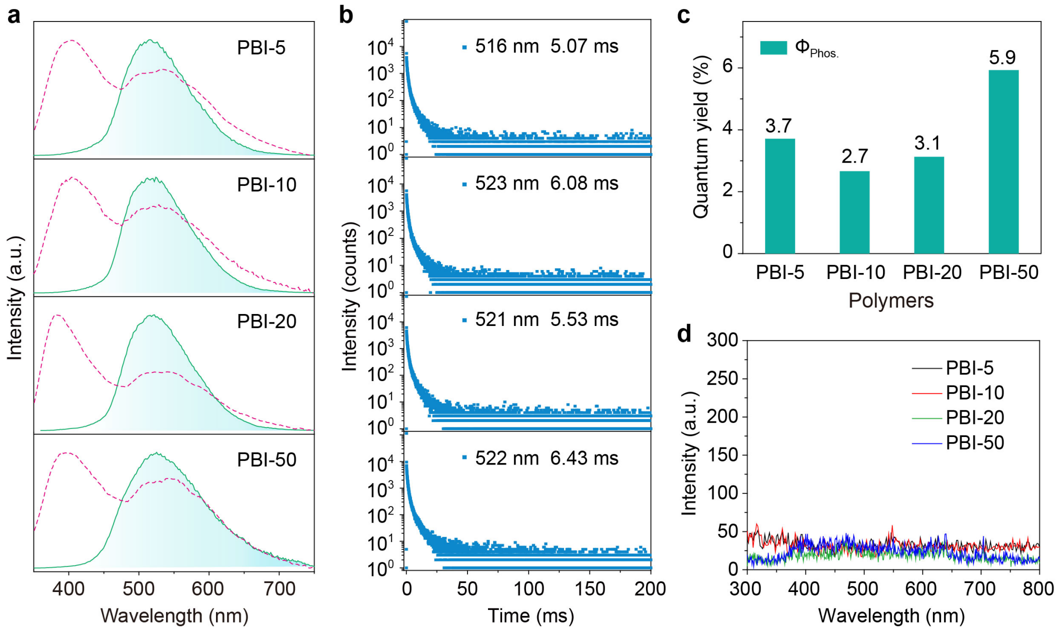

Supplementary Figure 39. **a** Normalized steady-state photoluminescence (red lines) and phosphorescence (green lines) spectra, **b** Lifetime profiles, and **c** phosphorescence efficiencies for PBI-5 to PBI-50 (the molar feed ratios of monomer BI/AA are 1/5, 1/10, 1/20 and 1/50) films, respectively. **d** RL spectra of different PBIs at a dose rate of 278 μGy s^{-1}.

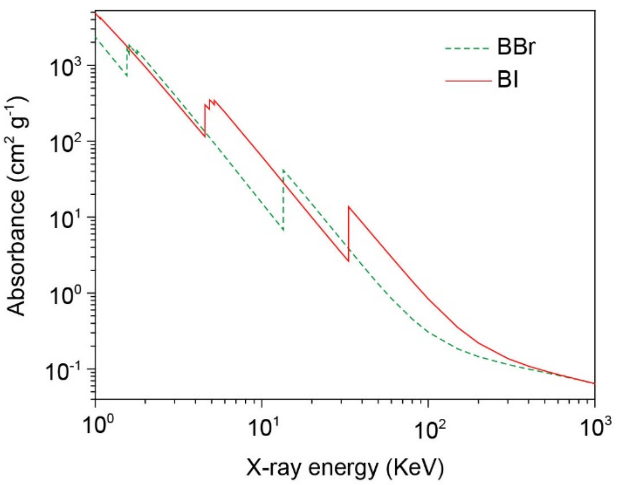

Supplementary Figure 40. X-ray absorption spectra of the BBr and BI monomers.

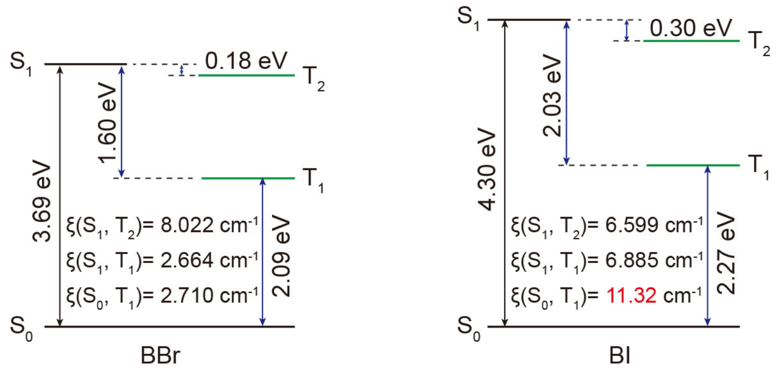

Supplementary Figure 41. Calculated excitation energies and spin-orbit coupling (SOC) constants (ξ) of the BBr and BI monomers. It was found that the ΔE_{ST} of BBr ($\Delta E_{S1\rightarrow T2}$ = 0.18 eV, $\Delta E_{S1\rightarrow T1}$ = 1.60 eV) was much smaller than that of BI ($\Delta E_{S1\rightarrow T2}$ = 0.30 eV, $\Delta E_{S1\rightarrow T1}$ = 2.03 eV), favoring the ISC process for generating triplet excitons. Meanwhile, for the ξ values of T_1 to S_0, monomer BI (S_0-T_1, 11.32 cm^{-1}) was much larger than BBr (S_0-T_1, 2.71 cm^{-1}), demonstrating a larger non-radiative decay for the triplet excitons of BI monomer (Supplementary Equations (7) and (8)).

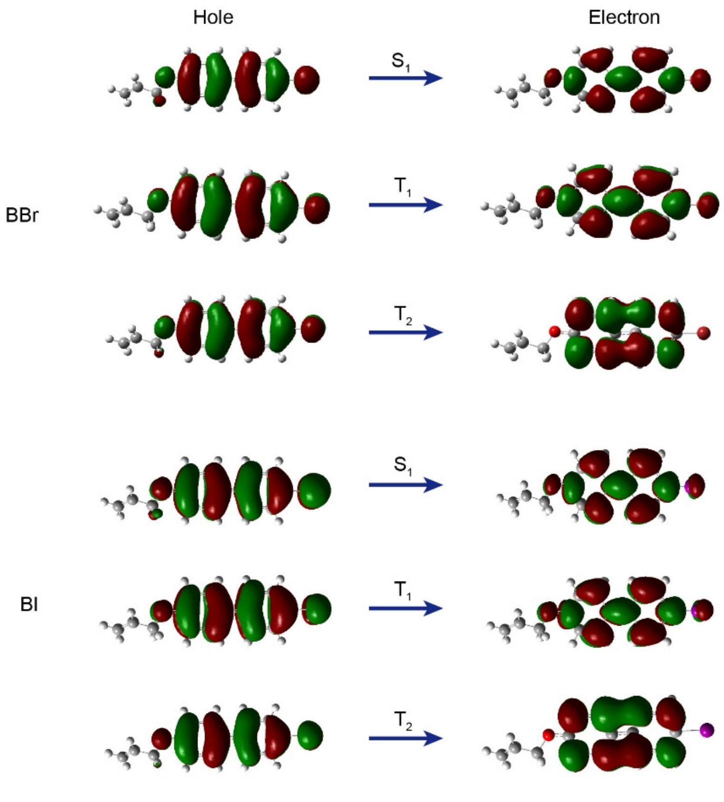

Supplementary Figure 42. Natural transition orbitals (NTOs) of monomers BBr and BI using the TD-DFT method.

The non-radiative decay rate k_{nr} is proportionate to the SOC between S_0 and T_1, which can be described as follows:[3]

$$k_{nr} = \sum_{m} \frac{2\pi}{\hbar} \left| \langle T_1^0 | \hat{\mathrm{H}}_{\mathrm{SOC}} | S_m^{\nu} \rangle \right|^2 \cdot FCWD' \quad (7)$$

$$FCWD' = \sum_{\nu} \exp(-S') \frac{S'^{\nu}}{\nu!} \cdot \frac{1}{\sqrt{4\pi\lambda' k_B T}} \exp\left[-\frac{(\Delta E' + \nu\hbar\omega + \lambda')^2}{4\pi\lambda' k_B T} \right] \quad (8)$$

Supplementary Table 14. Dynamic photophysical parameters of the phosphorescent copolymers.

Polymer	τ_f (ns)	τ_p (ms)	Φ_f (%)	Φ_p (%)	k_{isc} (s^{-1})	k_p (s^{-1})	k_{nr} (s^{-1})
PBI-5	4.67	5.07	5.4	3.7	2.03×10^8	7.71	189.5
PBI-10	3.98	6.08	3.7	2.7	2.42×10^8	4.61	159.9
PBI-20	5.78	5.53	5.5	3.1	1.63×10^8	5.93	149.6
PBI-50	5.55	6.43	5.3	5.9	1.71×10^8	9.69	145.8

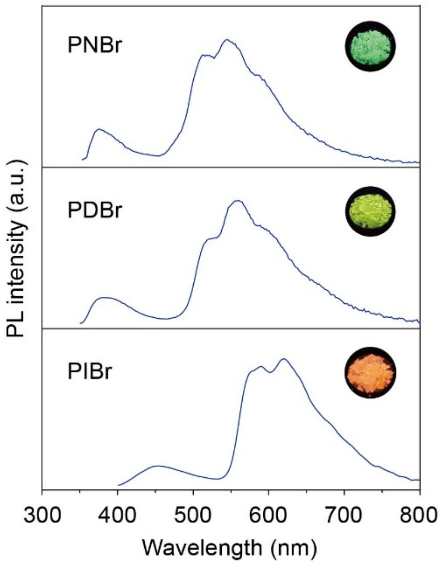

Supplementary Figure 43. Photoluminescence spectra and the related photographs of PNBr, PDBr, and PIBr copolymer films under UV irradiation.

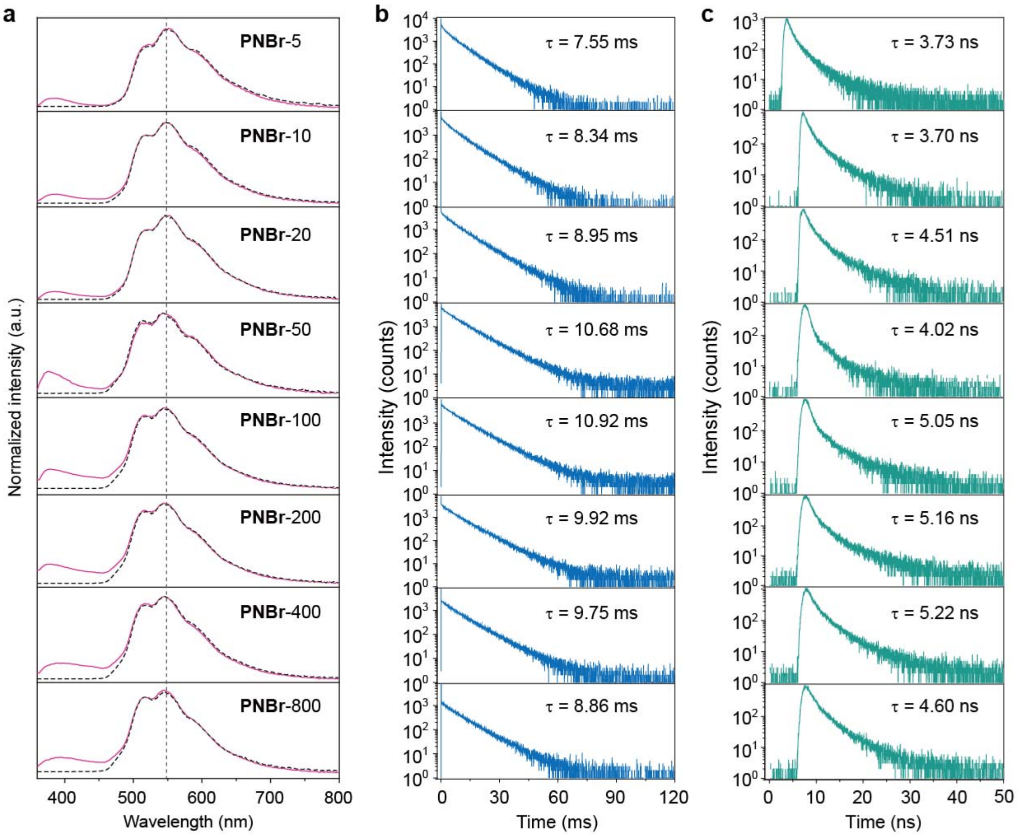

Supplementary Figure 44. **a**, **b**, and **c** Normalized steady-state photoluminescence (solid lines) and phosphorescence (dashed lines) spectra, and lifetime profiles of the emission bands at 545 and 380 nm for PNBr-5 to PNBr-800 films, respectively.

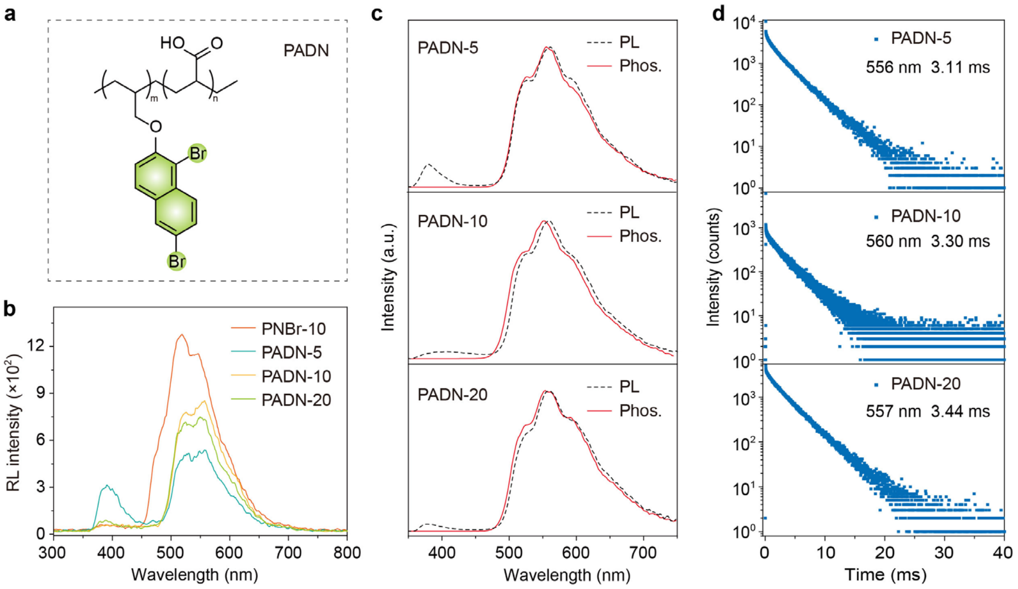

Supplementary Figure 45. **a** Chemical structure of the copolymer PADN. **b** RL spectra of the polymers PNBr-10 and PADN-5 to PADN-20 (the molar feed ratios of monomer ADN/AA are 1/5, 1/10, and 1/20) at a dose rate of 278 μGy s^{-1}. **c** Normalized steady-state photoluminescence (black lines) and phosphorescence (red lines) spectra of the copolymers PADN-5 to PADN-20 under ambient conditions. **d** Lifetime profiles of the emission band at around 560 nm for the copolymers PADN-5 to PADN-20.

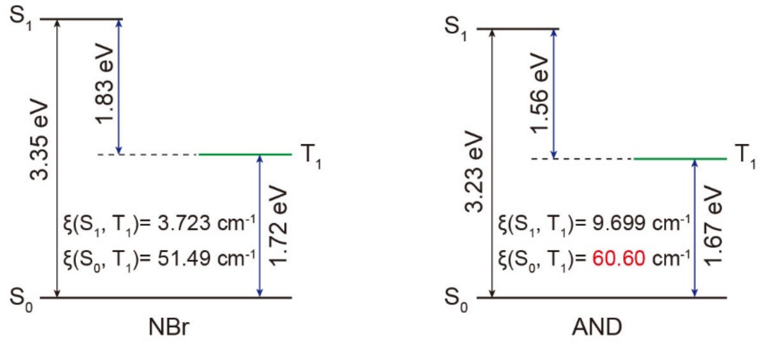

Supplementary Figure 46. Calculated excitation energies and spin-orbit coupling (SOC) constants (ξ) of the NBr and AND monomers. It was found that the spin-orbit coupling constants (ξ) of T_1 to S_0 for the monomer AND (60.60 cm^{-1}) was larger than that of NBr (51.49 cm^{-1}), demonstrating a larger non-radiative decay for the triplet excitons of AND monomer.

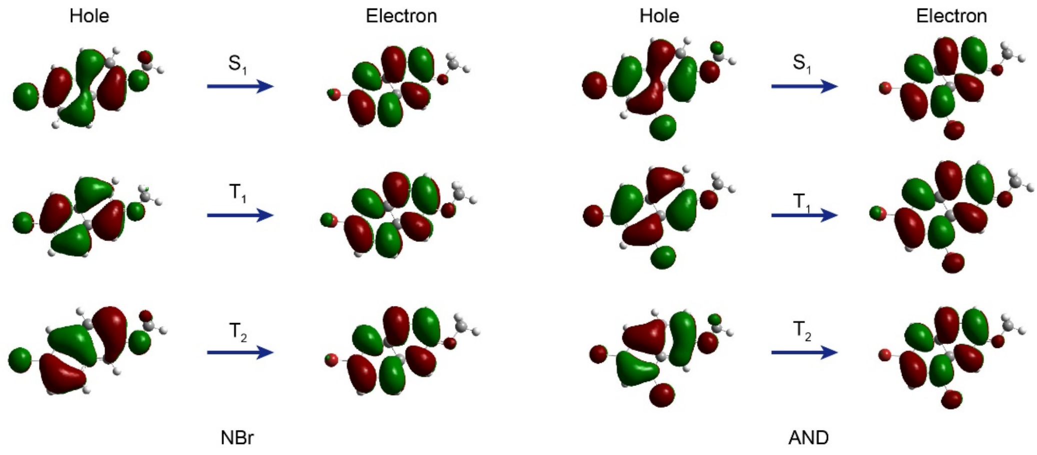

Supplementary Figure 47. Natural transition orbitals (NTOs) of the monomers NBr and AND using the TD-DFT method.

Supplementary Table 15. Dynamic photophysical parameters of the phosphorescent copolymers.

Polymer	τ_f (ns)	τ_p (ms)	Φ_f (%)	Φ_p (%)	k_{isc} (s^{-1})	k_p (s^{-1})	k_{nr} (s^{-1})
PNBr-10	3.70	8.34	1.1	15.2	2.67×10^8	18.4	101.4
PADN-5	4.33	3.11	0.3	3.8	2.30×10^8	12.3	309.3
PADN-10	4.03	3.30	0.5	10.2	2.47×10^8	31.1	271.9
PADN-20	4.68	3.44	0.3	13.5	2.13×10^8	39.4	251.3

From Supplementary Figures 37-42 and 45-47 and Supplementary Tables 13-15, both the absorption of X-ray and the decay of excited states of materials play a crucial role in obtaining the phosphorescence radioluminescence under X-ray irradiation. Actually, not any copolymers containing bromine-substituted chromophores and acrylic acid are possible for X-ray scintillation, and the organic dyes with high photoluminescence quantum yields are welcome. Meanwhile, although the X-ray absorptivity of materials could be promoted by the substitution of iodide or more bromine atoms on the monomers for generating excitons, the

large non-radiative decay of monomers would dissipate the populated triplet excitons and result in inefficient phosphorescence as well as radioluminescence. Therefore, simultaneously improving X-ray absorptivity as well as the ISC process and suppressing non-radiative transition of excitons favors to obtain the high-performance phosphorescence radioluminescence from purely organic polymer materials.

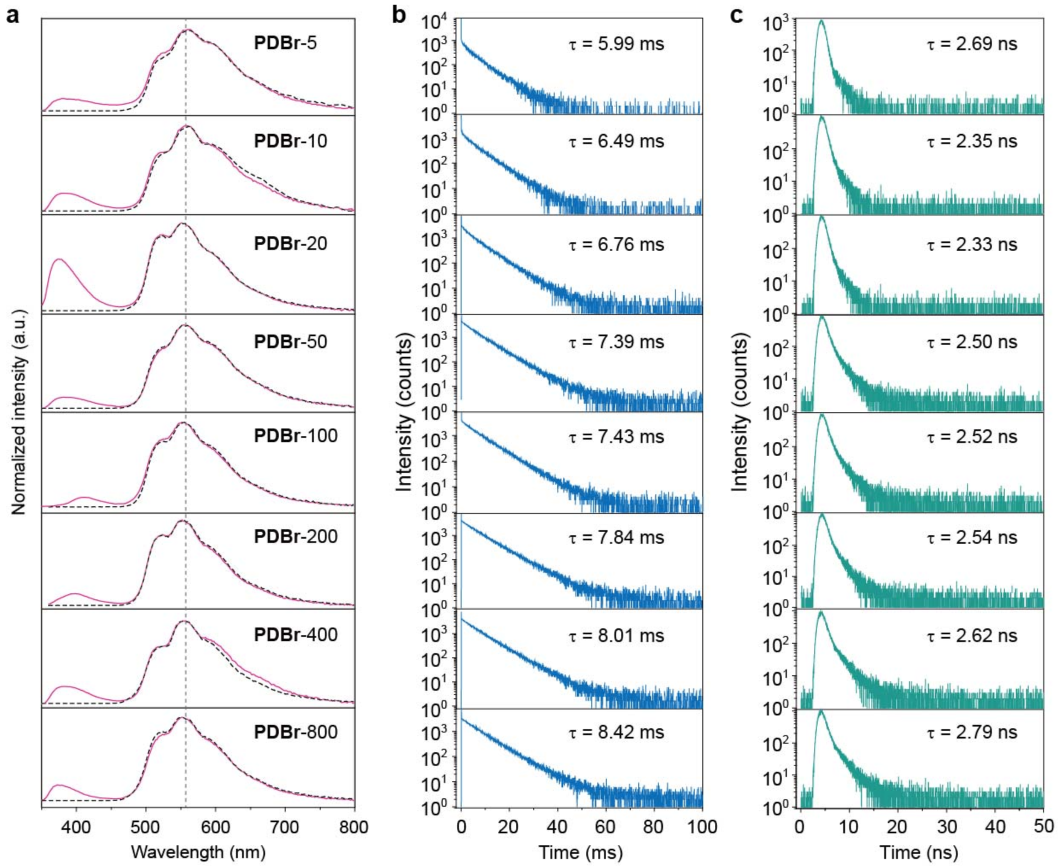

Supplementary Figure 48. **a**, **b**, and **c** Normalized steady-state photoluminescence (solid lines) and phosphorescence (dashed lines) spectra, and lifetime profiles of the emission bands at 553 and 370 nm for PDBr-5 to PDBr-800 films, respectively.

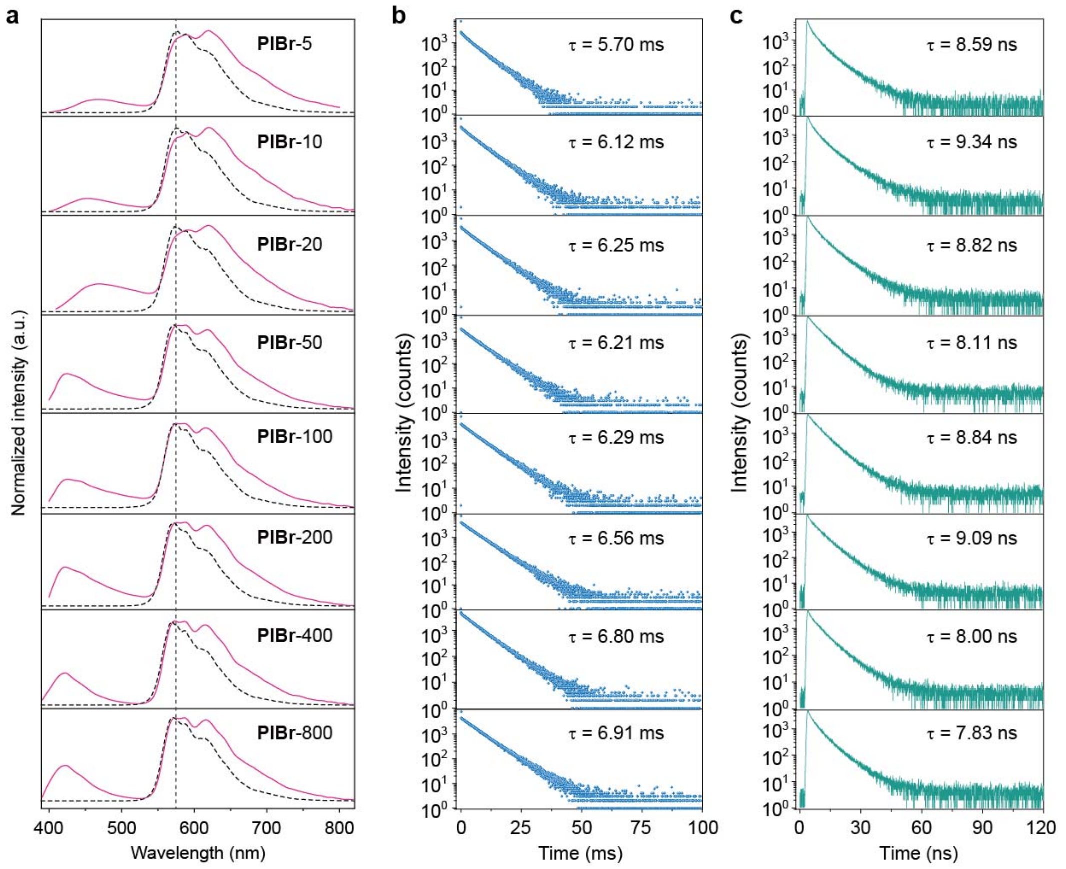

Supplementary Figure 49. **a**, **b**, and **c** Normalized steady-state photoluminescence (solid lines) and phosphorescence (dashed lines) spectra, and lifetime profiles of the emission bands at 575 and 430 nm for PIBr-5 to PIBr-800 films, respectively.

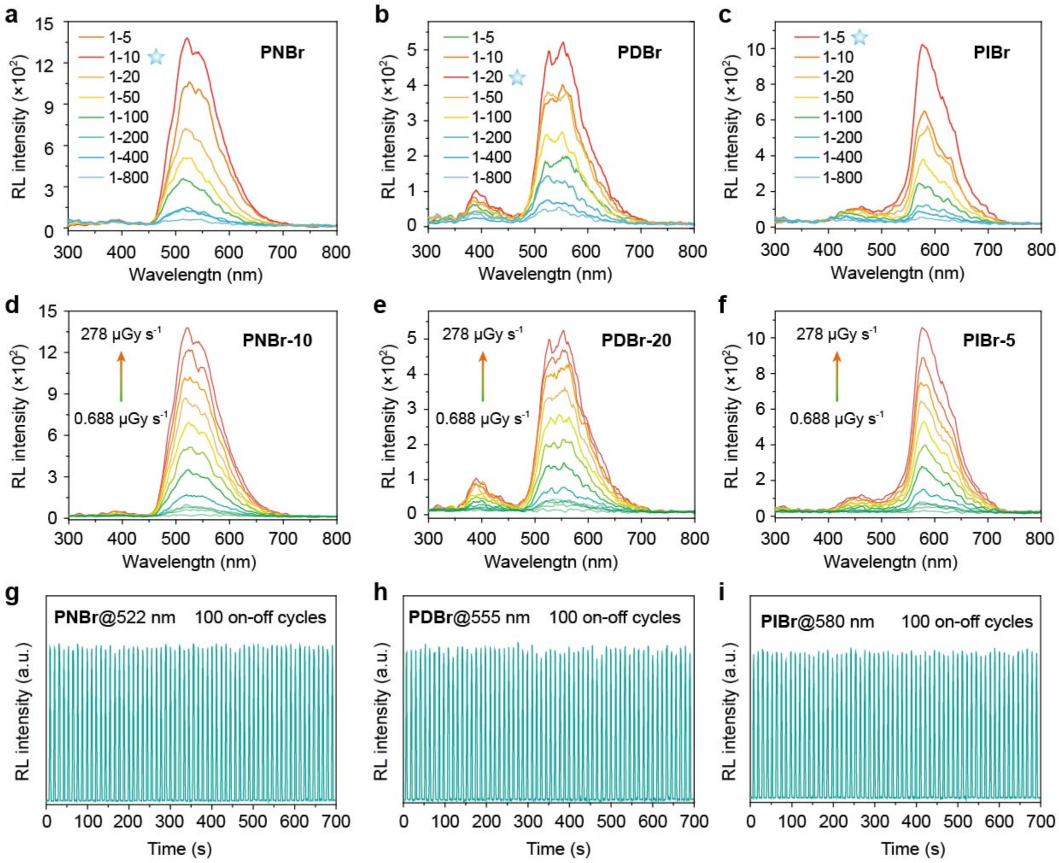

Supplementary Figure 50. X-ray-excited luminescence characterization of PNBr, PDBr, and PIBr, respectively. **a-c** RL spectra of polymers with different molar ratios at a dose rate of 278 μGy s^{-1}. Note: the examination was quantitative for each group. **d-f** The dosage dependent emission intensity in the range of 0.688 to 278 μGy s^{-1}. **g-i** The emission photostability of PNBr-10, PDBr-20 and PIBr-5 film under repeated on-off cycles of X-rays at a dose rate of 278 μGy s^{-1}.

Supplementary Table 16. Photoluminescence and phosphorescence quantum efficiencies of PNBr polymer films with various molar ratios of monomers under exposure of UV light.

Polymer	PNBr-5	PNBr-10	PNBr-20	PNBr-50	PNBr-100	PNBr-200	PNBr-400	PNBr-800
Φ_{PL} (%)	10.8	16.3	20.4	34.8	31.3	33.0	30.0	27.5
Φ_{Ph} (%)	10.1	15.2	19.2	30.8	29.1	30.7	27.9	25.6

Supplementary Table 17. Photoluminescence and phosphorescence quantum efficiencies of PDBr polymer films with various molar ratios of monomers under exposure of UV light.

Polymer	PDBr-5	PDBr-10	PDBr-20	PDBr-50	PDBr-100	PDBr-200	PDBr-400	PDBr-800
Φ_{PL} (%)	2.3	3.1	6.9	14.2	15.2	15.0	15.2	17.8
Φ_{Ph} (%)	2.1	2.9	5.4	13.7	14.7	14.2	14.5	17.1

Supplementary Table 18. Photoluminescence and phosphorescence quantum efficiencies of PIBr polymer films with various molar ratios of monomers under exposure of UV light.

Polymer	PIBr-5	PIBr-10	PIBr-20	PIBr-50	PIBr-100	PIBr-200	PIBr-400	PIBr-800
Φ_{PL} (%)	7.7	13.2	13.7	15.9	17.3	20.2	19.6	19.3
Φ_{Ph} (%)	7.0	12.5	10.1	12.5	14.4	15.8	17.1	16.5

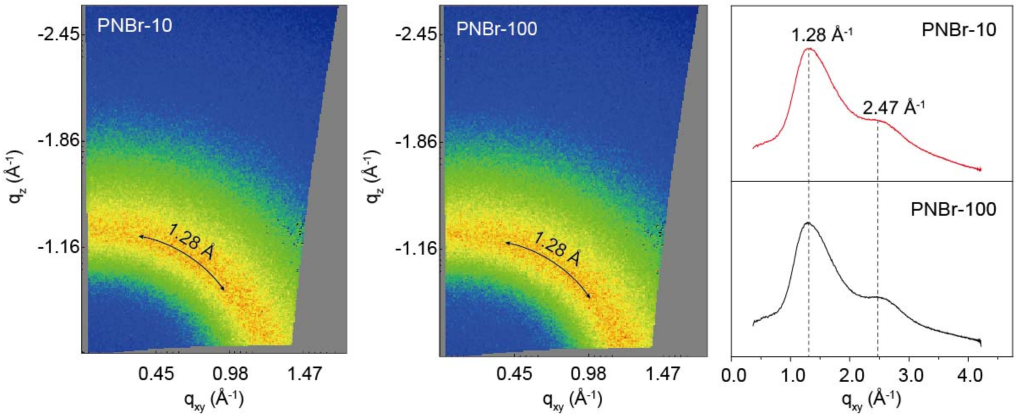

Supplementary Figure 51. Wide angle X-ray scattering patterns of polymer film PNBr-10 and PNBr-100.

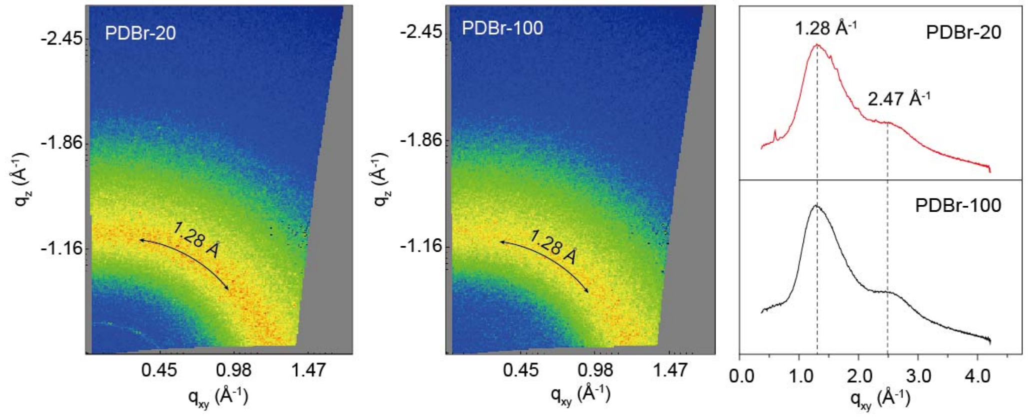

Supplementary Figure 52. Wide angle X-ray scattering patterns of polymer film PDBr-20 and PDBr-100.

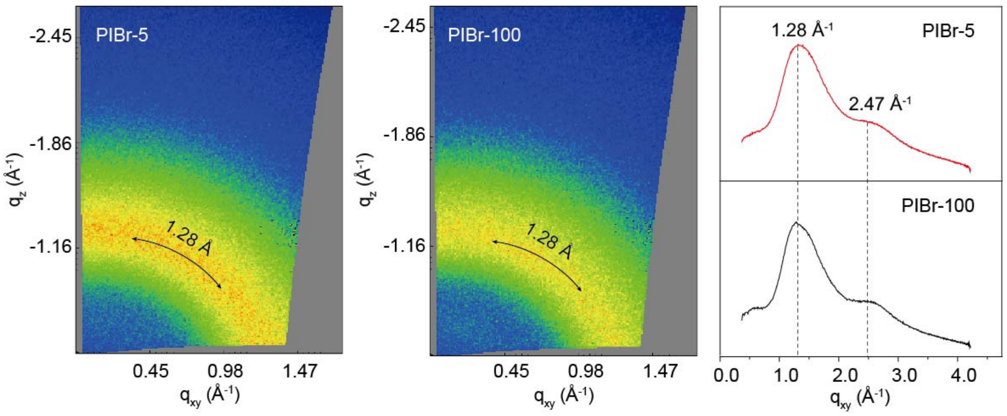

Supplementary Figure 53. Wide angle X-ray scattering patterns of polymer film PIBr-5 and PIBr-100.

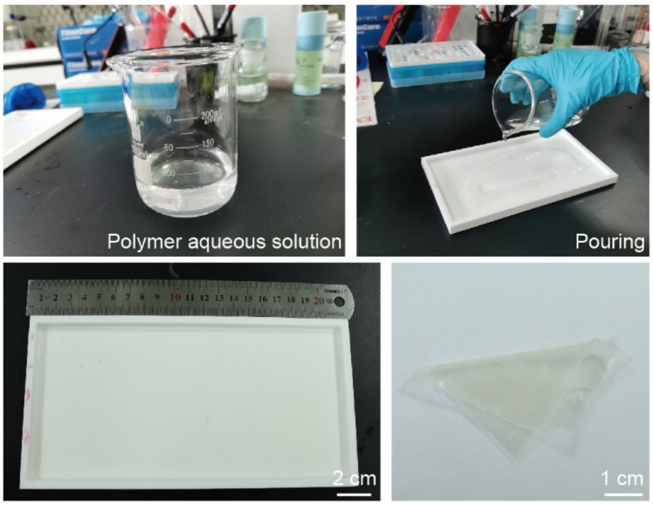

Supplementary Figure 54. Photographs of the preparation process of PNBr-10 film, and a uniform PNBr-10 film molded on a polytetrafluoroethylene mold and placed on a paper under day light.

Supplementary Table 19. The relationship between dose rate and voltage/current of the X-ray source.[1]

Voltage (kV)	Current (μA)	Dose rate (μGy s^{-1})
10	5	0.68844
20	5	4.5830
30	5	9.6410
40	5	11.912
50	5	17.375
50	10	34.750
50	20	69.500
50	30	104.25
50	40	139.00
50	50	173.75
50	60	208.50
50	70	243.25
50	80	278.00

Supplementary References

1. Wang, X. et al. Organic phosphors with bright triplet excitons for efficient X-ray-excited luminescence. *Nat. Photonics*. **15**, 187–192 (2021).

2. Gu, L. et al. Color-tunable ultralong organic room temperature phosphorescence from a multicomponent copolymer. *Nat. Commun.* **11**, 944 (2020).

3. Schmidt, K. et al. Intersystem crossing processes in nonplanar aromatic heterocyclic molecules. *J. Phys. Chem. A* **111**, 10490–10499 (2007).
