# Supplementary material for: Organic phosphorescent scintillation from copolymers by X-ray irradiation
Source: Nat Commun. 2022 Jul 9;13:3995. doi: 10.1038/s41467-022-31554-3 (PMC9271082; doi:10.1038/s41467-022-31554-3)
Supplement: Supplementary file 2 — Description of Additional Supplementary Files [file 41467_2022_31554_MOESM2_ESM.pdf]

Description of Additional Supplementary Files

File Name: Supplementary Movie 1
Description: Highly efficient room temperature phosphorescence of copolymer PBBr-50 under ambient conditions excited by a 310 nm lamp.
